# Alzheimer's disease diagnostic accuracy by fluid and neuroimaging ATN framework

**DOI:** 10.1111/cns.14357

**Published:** 2023-07-12

**Authors:** Xi Xiong, Haijun He, Qianqian Ye, Shuangjie Qian, Shuoting Zhou, Feifei Feng, Evandro F. Fang, Chenglong Xie

**Affiliations:** ^1^ Department of Neurology The First Affiliated Hospital of Wenzhou Medical University Wenzhou China; ^2^ Department of Clinical Molecular Biology Akershus University Hospital, University of Oslo Lørenskog Norway; ^3^ The Norwegian Centre on Healthy Ageing (NO‐Age) Oslo Norway; ^4^ Key Laboratory Of Alzheimer's Disease Of Zhejiang Province Wenzhou China; ^5^ Institute of Aging Wenzhou Medical University Wenzhou China; ^6^ Key Laboratory of Intelligent Treatment and Life Support for Critical Diseases of Zhejiang Province Wenzhou China

**Keywords:** ADNI, Alzheimer's disease, ATN biomarkers

## Abstract

**Objectives:**

The ATN's different modalities (fluids and neuroimaging) for each of the Aβ (A), tau (T), and neurodegeneration (N) elements are used for the biological diagnosis of Alzheimer's disease (AD). We aim to identify which ATN category achieves the highest potential for diagnosis and predictive accuracy of longitudinal cognitive decline.

**Methods:**

Based on the availability of plasma ATN biomarkers (plasma‐derived Aβ_42/40_, p‐tau181, NFL, respectively), CSF ATN biomarkers (CSF‐derived Aβ_42_/Aβ_40_, p‐tau181, NFL), and neuroimaging ATN biomarkers (18F‐florbetapir (FBP) amyloid‐PET, 18F‐flortaucipir (FTP) tau‐PET, and fluorodeoxyglucose (FDG)‐PET), a total of 2340 participants were selected from ADNI.

**Results:**

Our data analysis indicates that the area under curves (AUCs) of CSF‐A, neuroimaging‐T, and neuroimaging‐N were ranked the top three ATN candidates for accurate diagnosis of AD. Moreover, neuroimaging ATN biomarkers display the best predictive ability for longitudinal cognitive decline among the three categories. To note, neuroimaging‐T correlates well with cognitive performances in a negative correlation manner. Meanwhile, participants in the “N” element positive group, especially the CSF‐N positive group, experience the fastest cognitive decline compared with other groups defined by ATN biomarkers. In addition, the voxel‐wise analysis showed that CSF‐A related to tau accumulation and FDG–PET indexes more strongly in subjects with MCI stage. According to our analysis of the data, the best three ATN candidates for a precise diagnosis of AD are CSF‐A, neuroimaging‐T, and neuroimaging‐N.

**Conclusions:**

Collectively, our findings suggest that plasma, CSF, and neuroimaging biomarkers differ considerably within the ATN framework; the most accurate target biomarkers for diagnosing AD were the CSF‐A, neuroimaging‐T, and neuroimaging‐N within each ATN modality. Moreover, neuroimaging‐T and CSF‐N both show excellent ability in the prediction of cognitive decline in two different dimensions.

## Graphical abstract

The AUCs of CSF Aβ_42/40_ (CSF‐A), tau‐PET (neuroimaging‐T), and FDG–PET (neuroimaging‐N) were ranked the top three ATN candidates in diagnosis accuracy for AD. Neuroimaging ATN biomarkers demonstrated more strongly predictive targets of memory performances in terms of cognitive decline than CSF and plasma ATN categories. Neuroimaging‐T appeared to be an especially important correlation factor of memory function. We provide a detailed description and comprehensive analysis picture of the different sources of ATN biomarkers in the AD spectrum.

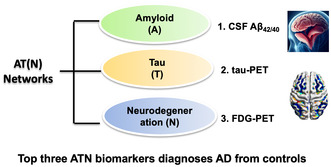

## 1 INTRODUCTION

By 2050, the morbidity of dementia will be tripled worldwide; for Alzheimer's disease (AD), the estimated numbers will be 3–4 times higher if based on the biological diagnosis than the numbers based on clinical symptoms.^1^ The earliest stage of the AD continuum happens in parallel with accumulated amyloid β (Aβ), leading to the propagation of tau pathology. Decades of efforts have been dedicated to the mechanistic studies of AD as well as to the development of sensitive and specific biomarkers for the early and easy diagnosis of AD, of the latter with challenges on replication and broad application.^2^, ^3^, ^4^ Novel multidimensional biomarkers contain PET scans and fluid assays for Aβ and phosphorylated tau (p‐tau), which show great prospects for clinical and research fields.^5^ Initially, AD confirmation was confined to the appearance of dementia, a clinical spectrum featured progressive cognitive decline, or neurobehavioral symptoms contributing to a low‐quality life. Of note, several studies displayed poor consistency between clinical diagnosis of probable AD and the existence of AD‐related pathologies at post‐mortem examination. The degree of diagnostic confidence was dissatisfactory partially due to the clinical symptoms of AD can be non‐specific and misleading.^6^

Because of biological characteristics and clinical feasibility, AD biomarkers are classified into three binary categories^7^: A (amyloid‐β deposition), T (tau accumulation: p‐tau), and N (neurodegeneration or neural injury), each characterized typically dichotomously as either negative (−) or positive (+) and called the ATN framework. The ATN system is a symptoms‐discreet, biomarkers‐driven classification scheme that categorizes individuals using multimodal biomarkers that chart core AD pathophysiological features.^8^ The “ATN” system offers a separate biological definition of AD and might be used to detect pathologic alterations that occur in both normal aging and AD dementia since the correct diagnosis of AD remains difficult for physicians.^9^ The existence of A (regardless of the presence of T and N) is termed Alzheimer's pathological change. Simultaneously, clinical phases can range from cognitively unimpaired‐to‐mild cognitive impairment (MCI) and dementia, emphasizing the continuum of AD stretches over a period of years. The ATN biological diagnostic framework underpins the importance of Aβ and p‐tau as the core characteristics of AD, hence promoting that AD can be diagnosed via biomarkers only, and even explicitly distinguishing it from other dementias, and as well could potentially be used in a precision medicine‐oriented method to give suitable treatments paradigm.^10^, ^11^ In terms of clinical practice, although the ATN approach is the cornerstone of disease‐modifying interventions in AD, there are still some unsuitable conditions, including operational difficulty in defining ATN positivity (+) or negativity (−), such as some biomarkers still lack widespread consensus cutoff values, and also different biomarkers, different sources (e.g., cerebrospinal fluid (CSF), plasma‐based and neuroimaging) being incorporated into one category.^10^

Deep‐excavated CSF and neuroimaging ATN biomarkers systems showed, with a trend that is not yet fully established, fluid biomarkers showed meaningful change earlier than neuroimaging biomarkers. Reduced Aβ_42_ levels and the Aβ_42_/Aβ_40_ ratio in the CSF are early readouts of Aβ dyshomeostasis compared to the Aβ–PET.^12^ Recently, a particularly exciting biomarker direction of AD is substantial evidence has been made in blood‐based biomarkers development for AD.^13^, ^14^ The ATN elements are likely to update new blood biomarkers for Aβ, tau pathology, neurodegeneration, and other pathophysiological features (X) as a first‐tier screening tool or still not yet available. For instance, CSF or blood neurofilament light chain (NFL) levels as a proxy of the “N” element can provide an index of whether some interventions halt neuronal loss.^15^ Although the different modalities (fluids and neuroimaging) for each of the Aβ, tau, and neurodegeneration elements have been tested for clinical use and are considered interchangeable,^16^, ^17^, ^18^, ^19^ concordance between the fluids and neuroimaging ATN biomarkers has recently been challenged. Moreover, the novelty of the current report, in comparison with the previous reports, aims at comprehensive evaluation the discrepancies between CSF, neuroimaging, and blood ATN modalities for patients from normal, MCI, and AD. Nevertheless, uncertainty regarding the order of diagnostic accuracy between the different types of sources hinders the widespread implementation of these ATN biomarkers, and whether blood tests can replace CSF tests or neuroimaging. There is, therefore, now a need to identify which ATN category or tissue achieves the highest potential for early diagnosis and predictive accuracy of longitudinal cognitive decline. Moreover, other nonetheless of the utmost important objective of this project is to determine if a patient with subtle cognitive decline symptoms, suffers from prodromal AD, and will progress to AD dementia within the near future can be captured through ATN biomarkers, and define accepted flexible cut‐off points for these biomarkers.

## 2 METHODS

### 2.1 Study design

We used the STARD reporting guidelines for this study.^20^ Data were acquired from the Alzheimer's Disease Neuroimaging Initiative (ADNI) public database (http://adni.loni.usc.edu). The ongoing ADNI was founded in 2003 as a public–private partnership, led by Principal Investigator Michael W. Weiner, MD. The main goal of ADNI has been to test whether serial neuroimaging, fluid biological markers, and clinical neuropsychological assessment can be combined to predict the progression of MCI and early AD from 57 sites in the USA and Canada. Specifically, data were downloaded from the ADNI dataset since these data contained cognitive test results, including memory composite score (ADNI–MEM), executive functioning composite score (ADNI–EF), and Alzheimer's Disease Assessment Scale–Cognitive Subscale (ADAS–Cog)^21^ for the present study. A total of 2340 participants were selected from ADNI based on the availability of plasma ATN biomarkers (plasma‐derived Aβ_42_/Aβ_40_, p‐tau181, NFL), CSF ATN biomarkers (CSF‐derived Aβ_42/40_, p‐tau181, NFL) and neuroimaging ATN biomarkers (18F‐florbetapir (FBP), 18F‐flortaucipir (FTP) tau‐PET, and FDG–PET), respectively. It has been suggested that the Aβ_42/40_ ratio is superior to Aβ_42_ alone in predicting Aβ deposition,^22^ hence we choose Aβ_42/40_ as the “A” component. The two single‐nucleotide polymorphisms for ApoE (rs429358, rs7412) were genotyped using DNA extracted by Cogenics from a 3 mL aliquot of EDTA blood. Participants were assigned to the ApoE ε4 carriers, defined as individuals carrying at least one ε4 allele.

### 2.2 Participants' characteristics and patient consent

Participants from the ADNI cohort consisted of consecutively included cognitively normal (CN: MMSE >24, CDR = 0) subjects, with mild cognitive symptoms (MCIs: MMSE >24, CDR = 0.5) and AD dementia (19 < MMSE < 24, CDR = 0.5–1) who were diagnosed by ADNI centers and referred to the participating memory clinics. The patients were divided into having either CN, MCI, or AD according to the extensive neuropsychological battery performed at baseline, including verbal, episodic memory, visuospatial ability, and attention/executive domains. In concordance with the Diagnostic and Statistical Manual of Mental Disorders, 5th Edition (DSM‐5) criteria for MCI, all participants with composite z‐scores of −1 to −1.5 were individually assessed by a senior neuropsychologist and established as having MCI if the performance was evaluated to show an obvious cognitive decline when compared with their estimated premorbid level. The participants were followed longitudinally with yearly included cognitive testing by experienced physicians in neurocognitive fields and measurements of the biomarkers. All patients, or their partners, gave written informed consent to participate at each site, and the study was approved by the institutional review boards of all participating institutions and ethics committees.

### 2.3 Neuropsychological assessment and clinical progression prediction

Brief cognitive tests in ADNI were selected to represent different cognitive domains. ADNI assessed the cognitive status of participants annually using the 11‐ and 13‐point versions of the ADAS‐11 and ADAS‐13.^21^ Of note, the ADNI neuropsychological test battery contains multiple indicators for memory functions, on which ADNI–MEM has been established. ADNI–MEM is a composite score formed from the Rey Auditory Verbal Learning task, word list learning and recognition tasks from ADAS‐Cog, recall from Logical Memory I of the Wechsler Memory Test‐Revised, and the 3‐word recall item from the MMSE.^23^ Moreover, ADNI–EF is constitutive of Category Fluency (i.e., animals and vegetables), the Trail‐Making Test parts A and B, Digit Span Backwards, Wechsler Adult Intelligence Scale‐Revised Digit–Symbol Substitution, and 5 Clock Drawing items.^24^ The ten‐word delayed recall test from the ADAS–Cog has been validated for detecting early cognitive decline. The naming objects and fingers task from the ADAS–Cog was used for verbal performance, and the clock‐drawing test was used for visuospatial performance. Each domain was converted to a *z*‐score based on the test score distribution in the present population. In addition, the MMSE was used as a brief test of global cognition with specific sensitivity to the cognitive decline seen in AD.^25^ The main outcome was the prediction of progression to dementia within a follow‐up of 4 years.

### 2.4 Plasma and CSF biomarkers measurements

Blood samples were collected at baseline and analyzed according to the standardized ADNI protocol.^26^ Plasma concentrations of Aβ_42_ and Aβ_40_ were measured using Module A of the INNO–BIA plasma Aβ forms immunoassay kit (Innogenetics, Ghent, Belgium, for research use‐only reagents) on the Luminex 100 immunoassay platform and IS v.2.3 software (Luminex).^27^, ^28^ Plasma p‐tau181 and NFL in ADNI were measured on Simoa HD‐X instruments (Quanterix) at the Clinical Neurochemistry Laboratory, the University of Gothenburg, according to previous papers.^29^, ^30^ The capture mouse antibody AT270 (MN1050, Invitrogen), specific for the p‐tau 181 site, was coated onto paramagnetic beads (103,207, Quanterix), and the detector antibody tau12 (806,502, BioLegend) was biotinylated. These reagents were used together with recombinant tau 441 phosphorylated in vitro by glycogen synthase kinase 3β (TO8‐50FN, SignalChem) as the calibrator to build the assay. Longitudinal blood sampling was tested approximately every year, over a median follow‐up time of 2.9 years. CSF samples were collected and processed based on previously described protocols.^26^ Levels of Aβ_42_, Aβ_40,_ and p‐tau181 in CSF were measured by the ADNI Biomarker Core using the Elecsys immunoassay.^31^ Note that absolute Aβ_42,_ Aβ_40,_ and p‐tau181 levels in CSF, as determined by the Elecsys assay, are not comparable with those tested in plasma by the Simoa assay as the assays use different antibodies and calibrators. CSF NFL levels were determined by a commercial, sensitive sandwich ELISA method.^32^

### 2.5 Neuroimaging acquisition, PET, and MRI preprocessing

All the imaging data were downloaded from the ADNI–LONI and IDA image archive (https://ida.loni.usc.edu). ADNI MRI and PET acquisition protocols are detailed elsewhere, see www.adni‐info.org. PET scans were acquired according to published protocols and analyzed using tracer‐specific acquisition windows: 50–70 min for 18F‐florbetapir (FBP), 75–105 min for FTP, and 30–60 min for FDG. The mean tracer uptake of selected cortical and reference regions was calculated with the PET scan applied to its corresponding MRI scan. The scans were already averaged, aligned to standard space, resampled to a standard image and voxel size (2 mm × 2 mm × 2 mm), and smoothed to a uniform resolution as previously described.^33^ We aligned the images to the corresponding MRI scan from the same visit and normalized them to MNI space using parameters obtained from the MRI segmentation using Statistical Parametric Mapping version 12 (SPM12; Wellcome Trust Center for Neuroimaging, London, UK, http://www.fil.ion.ucl.ac.uk/spm). Summary FBP standard uptake value ratios (SUVRs) were generated by averaging uptake ratios across four cortical regions (frontal, anterior cingulate, precuneus, and parietal cortex). A composite metaROI (region of interest) of bilateral entorhinal, amygdala, fusiform, parahippocampal, inferior, and middle temporal regions were considered for tau PET assessment. The average counts of FDG–PET across angular, temporal, and posterior cingulate regions were adopted. Reference regions used mirrored published papers (pons for FDG, whole cerebellum for amyloid, and cerebellar crus for tau PET).^34^, ^35^ A total of 259 different brain regions were obtained by MRI classification using the multi‐atlas labeling method. The pre‐processed T1 scanned image data (GradWarp, B1 calibrated, N3, Scaled) were downloaded from ADNI, further brain extraction (skull removal) was performed,^36^ and a novel multi‐atlas labeling method based on atlas registration was used to segment the regions of interest.^37^ The software can be obtained from the website https://www.cbica.upenn.edu/sbia/software/index.html. The resulting volume data for each brain region can be downloaded from the ADNI dataset. Among the different MRI brain regions obtained by a novel multi‐atlas labeling method, the left hippocampus was chosen as the representative brain region of MRI through the rank of importance percentage obtained by the Random Forest model for CN/AD classification (Figure S1).

### 2.6 Statistical analysis

Statistical analysis was performed using R software (Version 4.1.0), Statistical Parametric Mapping version 12 (SPM12), MedCalc software (Version 19.2.6), and SPSS (Version 26). Continuous variables were assessed for normality using the Kolmogorov–Smirnov test and the Quantile–Quantile Plot. Approximately normally distributed variables were expressed as the mean ± standard deviation (SD). A two‐sample *t*‐test was used for comparison between two groups, and a one‐way analysis of variance was used for comparison between multiple groups. Non‐normally distributed were expressed as the median [25th%, 75th%]. The Mann–Whitney U rank sum test was used for comparison between two groups, and the Kruskal–Wallis test was used for comparison between multiple groups. Categorical variables were presented as numbers (percentages) and were compared using the Chi‐square test or Fisher's exact test. Pairwise comparison was performed by Bonferroni correction post hoc test. Two‐tail tests showed a statistically significant difference at *p* < 0.05.

The Random Forest method was used to sort the characteristics of CN/AD discrimination by the volume of each brain region, and the brain region with the best CN/AD discrimination ability was obtained as the representative biomarker of structural MRI Brain region volume. Receiver–operating characteristic (ROC) curves were used to quantify the area under the ROC curve (AUC). The final AT(N) framework biomarkers were determined by comparing the area AUC (Delong test) under the ROC curve of the AT(N) groups of biomarkers from different sources to distinguish CN/AD by MedCalc software. Threshold values for ATN fluid biomarkers and FDG–PET were calculated based on the Youden index, using ROC analyses. For amyloid PET, higher than 1.11 SUVR was considered to have abnormal cortical amyloid deposition (PET‐A+).^38^ For tau PET, FTP SUVRs without correction for partial volume effects were calculated, and SUVR higher than 1.25 was considered to have tau pathology (PET‐T+).^39^

We assessed linear associations of baseline multidimensional ATN biomarker levels with cross‐sectional and longitudinal estimates of cognitive functions as measured by neuropsychological assessment. Longitudinal annual changes in cognitive tests were calculated using linear mixed‐effects models (LMM) with 4‐year cognitive test results. LMM had cognitive test scores as the dependent variable and included the independent variables' time (years between baseline and follow‐up time points) for fixed effects and random effects, adjusted for sex, age, and years of education. The Spearman correlation coefficient was calculated by the covariance of two variables over the product of their standard deviation. The value range of the Spearman correlation coefficient is from −1 to 1, with a higher absolute value indicating a stronger association and the sign indicating a positive or negative association between the two variables. The relationship between baseline scores or longitudinal annual changes in cognitive tests and ATN biomarker values, including biomarker levels (natural log‐transformed and standardized due to non‐normal distribution) and positive or negative ATN status, was estimated to investigate whether baseline ATN biomarkers values can predict subsequent 4‐year cognitive trajectory. We further assessed associations of baseline CSF Aβ_42/40_ level with baseline and 4‐year‐later cerebral tau pathology and glycometabolism measured on tau‐PET and FDG–PET for different diagnostic groups, using linear voxel‐wise regressions adjusted for age and sex. Neuroimaging analyses were performed using SPM12. Multivariate logistic models were used to construct the diagnostic model. Each multivariate logistic model implied a combination of one biomarker of “A”, one biomarker of “T”, and one biomarker of “N”. The predictive value of each model was calculated and the AUC stood as a measure of discriminating between normal and AD subjects.

## 3 RESULTS

### 3.1 Participants in the ADNI database

Participant's data were extracted from the ADNI database, and the basic demographic characteristics are shown in Table 1. A total of 2340 patients were selected, comprising 863 individuals with normal cognition (or CN), 1068 clinically diagnosed with MCI, and 409 patients with AD. Participants were required to have CSF, plasma, or neuroimaging ATN biomarkers in the analysis. The mean age of patients with CN and MCI (72.7 and 72.8 years, respectively) was significantly lower than that of AD participants (74.9 years). There were more female subjects in AD and MCI (56.4% of AD and 58.6% of MCI) compared to CN (44.3%), as well as low‐education levels in the AD group. Participants carrying the APOE ε4 allele, the largest genetic risk factor for AD, were particularly overrepresented in AD and MCI groups (67.0% vs. 49.7%, respectively) compared with CN (30.4%). As indicated by MMSE, CDR (clinical dementia rating), ADAS‐11, ADAS‐13, and ADNI‐MEM scores et al., cognitive tests significantly differed between dementia groups and controls. To compare the diagnostic accuracy of AT(N) biomarkers between the different types of sources (fluid and neuroimaging ATN framework), a novel AT(N) biomarker framework was constructed, which contains the optimum biomarkers in each AT(N) dimension. The differences in AUC values among biomarkers from the same source were further compared by the Delong test, as shown in Table S1. According to the AUC values, the optimal framework biomarkers of AT(N) were finally selected, as shown in Table S2.

**TABLE 1:** Baseline demographics and ATN data of participants with ADNI.

	CN (*N* = 863)	MCI (*N* = 1068)	AD (*N* = 409)	*p* value (CN vs. MCI)	*p* value (CN vs. AD)	*p* value (MCI vs. AD)
ADNI‐1	229 (26.5%)	397 (37.2%)	192 (46.9%)	–	–	–
ADNI‐GO	295 (34.2%)	341 (31.9%)	150 (36.7%)	–	–	–
ADNI‐2	338 (39.2%)	201 (18.8%)	67 (16.4%)	–	–	–
ADNI‐3	1 (0.12%)	129 (12.1%)	0 (0.00%)	–	–	–
Age (years)	72.7 (6.35)	72.8 (7.64)	74.9 (7.91)	0.927	<0.001	<0.001
Gender (% female)	382 (44.3%)	626 (58.6%)	230 (56.2%)	<0.001	<0.001	0.492
Educaiton (years)	16.0 [13.0;18.0]	16.0 [15.0;18.0]	16.0 [14.0;18.0]	<0.001	<0.001	<0.001
APOE4 carriers (%)	246 (30.6%)	503 (49.7%)	262 (67.0%)	<0.001	<0.001	<0.001
CSF ATN biomarkers levels						
CSF_Aβ_40_ (pg/mL)	18,320 [15,110;21,970]	17,490 [13,880;21,480]	16,165 [12,258;18,558]	0.180	0.002	0.022
CSF_Aβ_42_ (pg/mL)	1136 [815;1629]	752 [564;1271]	544 [424;714]	<0.001	<0.001	<0.001
CSF_Aβ_42/40_	0.07 [0.05;0.09]	0.05 [0.03;0.08]	0.04 [0.03;0.04]	<0.001	<0.001	<0.001
CSF p‐tau181 (pg/mL)	19.8 [14.9;26.6]	24.8 [18.8;36.0]	32.3 [26.5;47.5]	<0.001	<0.001	<0.001
CSF T‐tau (pg/mL)	226 [173;293]	263 [212;356]	331 [267;432]	<0.001	<0.001	0.001
CSF_NFL (pg/mL)	1044 [810;1263]	1320 [1020;1693]	1479 [1152;1841]	<0.001	<0.001	0.012
Plasma ATN biomarkers levels						
Plasma_Aβ_40_ (pg/mL)	155 [122;183]	153 [122;184]	154 [129;178]	0.959	0.959	0.959
Plasma_Aβ_42_ (pg/mL)	37.8 [29.8;45.0]	35.5 [27.9;43.9]	37.2 [30.6;42.2]	0.347	0.508	0.508
Plasma_Aβ_42/40_	0.25 [0.22;0.29]	0.24 [0.20;0.29]	0.24 [0.21;0.27]	0.322	0.322	0.879
Plasma p‐tau181 (pg/mL)	14.0 [9.85;19.2]	17.1 [11.2;24.4]	23.0 [17.5;27.8]	<0.001	<0.001	<0.001
Plasma T‐tau (pg/mL)	2.52 [1.77;3.11]	2.62 [1.76;3.45]	2.82 [2.09;3.84]	0.218	0.002	0.045
Plasma_NFL (pg/mL)	30.4 [23.8;41.1]	35.9 [26.6;49.0]	44.5 [33.6;59.0]	<0.001	<0.001	<0.001
Neuroimaging ATN biomarkers levels						
PET_SUVR (Aβ)	1.23 [1.13;1.39]	1.31 [1.17;1.59]	1.59 [1.37;1.79]	<0.001	<0.001	<0.001
PET_SUVR (tau)	1.18 [1.13;1.23]	1.22 [1.15;1.37]	1.53 [1.27;1.80]	<0.001	<0.001	<0.001
PET_SUVR (FDG)	1.28 [1.20;1.36]	1.22 [1.12;1.31]	1.04 [0.94;1.13]	<0.001	<0.001	<0.001
L‐HV (mm^3^)	3540 (414)	3203 (491)	2888 (501)	<0.001	<0.001	<0.001
Cognitive tests						
ADAS‐11	5.33 [3.67;7.33]	9.67 [7.00;13.0]	19.0 [14.7;23.0]	<0.001	<0.001	<0.001
ADAS‐13	29.0 [29.0;30.0]	28.0 [26.0;29.0]	23.0 [21.0;25.0]	<0.001	<0.001	<0.001
MMSE	1.02 (0.57)	0.19 (0.67)	−0.86 (0.53)	<0.001	<0.001	<0.001
ADNI‐MEM	0.88 (0.82)	0.22 (0.89)	−0.90 (0.96)	<0.001	<0.001	<0.001
ADNI‐EF	5.33 [3.67;7.33]	9.67 [7.00;13.0]	19.0 [14.7;23.0]	<0.001	<0.001	<0.001

*Note*: Approximately normally4 distributed variables were expressed as the mean ± standard deviation (SD). One‐way analysis of variance was used for comparison between multiple groups. Non‐normally distributed were expressed as the median [25th percent, 75th percent] and the Kruskal–Wallis test was used for comparison between multiple groups. Categorical variables were presented as numbers (percentages) and were compared using the Chi‐square test. Pairwise comparison was performed by Bonferroni correction post hoc test.

Abbreviations: AD, Alzheimer's disease; ADAS, Alzheimer's disease assessment scale‐cognitive subscale; ADNI‐EF, composite executive functioning score; ADNI‐MEM, composite memory score; Aβ, amyloid beta; CDRSB, clinical dementia rating sum of boxes; CN, cognitively normal; CSF, Cerebro‐spinal Fluid; FDG, fluorodeoxyglucose; L‐HV, Left hippocampal volume; MCI, mild cognitive impairment; MMSE, mini‐mental state examination; NA, not applicable; NFL, neurofilament light; PET, positron emission tomography; p‐tau, phosphorylated tau 181; SUVR, standardized uptake value ratio; T‐tau, total tau.

**FIGURE 1:**
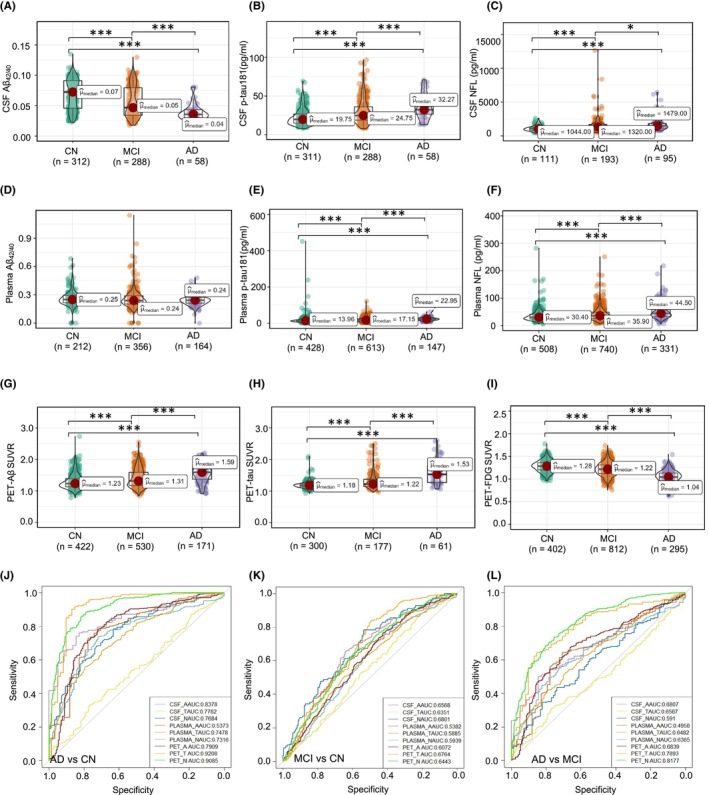
CSF, plasma, and neuroimaging ATN biomarkers profiles. Distribution of CSF (A–C), plasma (D–F), and neuroimaging (G–I) ATN biomarkers concentrations across the separate clinically defined diagnostics groups, namely CN, MCI, and AD. The different sample sizes of different markers are also displayed at the bottom of the figures. Comparing CSF, plasma, and neuroimaging ATN biomarkers diagnosis accuracy in AD versus CN (J), MCI versus CN (K), and AD versus MCI (L) comparisons using receiver operating characteristic (ROC) curve analyses. Statistical differences among the groups for each biomarker were determined by the Kruskal–Wallis test followed by multiple comparisons with Holm–Bonferroni adjusts (A–I).

In addition, to determine the order of diagnostic accuracy of AD between the different types of sources (fluid and neuroimaging ATN framework), the levels of CSF, plasma, and neuroimaging ATN biomarkers found in the different diagnostic groups are also shown in Table 1. In accordance with previous reports,^40^ baseline CSF Aβ_1–40_, CSF Aβ_1–42,_ and CSF Aβ_42/40_ ratio (CSF‐A classification) levels were significantly lower in AD dementia (CSF Aβ_42/40_: 0.04 [0.03;0.04]) and MCI (CSF Aβ_42/40_: 0.05 [0.03;0.08]) as compared to the CN group (CSF Aβ_42/40_: 0.07 [0.05;0.09]; Figure 1A and Table 1). Regarding the CSF‐T classification, the higher CSF p‐tau181 concentration in AD dementia (32.3 [26.5;47.5] pg/mL) as compared to the MCI (24.8 [18.8;36.0] pg/mL) and CN (19.8 [14.9;26.6] pg/mL) was highly significant (*p* < 0.001; Figure 1B). Of note, there was an upward trend of higher CSF NFL (CSF‐N classification) in the AD (1479 [1152;1841] pg/mL) and MCI (1320 [1020;1693] pg/mL) compared with the CN group (1044 [810;1263] pg/mL; Figure 1C). The use of CSF biomarkers is still limited because of high costs, hard availability, and invasive traits. There is, therefore, a great interest in plasma‐based ATN biomarkers. Within the plasma ATN biomarkers, there was no difference in terms of Plasma‐A among each group (*p* > 0.05, Figure 1D), and plasma p‐tau181 (Plasma‐T) was higher in participants classified as AD and MCI compared to those determined as CN (*p* < 0.001, Figure 1E). When considering Plasma‐N classification, plasma NFL was obviously higher in the AD and MCI groups than in the CN group (*p* < 0.001, Figure 1F). To test the neuroimaging ATN biomarkers in the AD continuum, Aβ–PET SUVR (neuroimaging‐A) and tau‐PET SUVR (neuroimaging‐T) were both higher in the AD dementia compared with the CN group (*p* < 0.001, Figure 1G,H), whereas FDG–PET SUVR (neuroimaging‐N) was significantly lower in the AD and MCI than in the CN group (*p* < 0.001, Figure 1I).

### 3.2 Discriminative accuracy of CSF, plasma, and neuroimaging ATN biomarkers for AD patients in the ADNI cohort

To test the diagnostic accuracy of CSF, plasma, and neuroimaging ATN biomarkers in distinguishing clinically defined diagnostic groups. For the primary outcome of AD veraus CN (Figure 1J), the AUC was 0.8378 using the CSF Aβ_42/40_ ratio (CSF‐A), which was significantly higher than the AUCs for plasma Aβ_42/40_ ratio (Plasma‐A: AUC 0.5373) and Aβ–PET (neuroimaging‐A: AUC 0.7909), indicating the CSF‐A classification is the best candidate to differentiate the AD from CN in the “A” element. Similarly, regarding the “T” element in the ATN framework, the AUC for tau‐PET (neuroimaging‐T) levels was 0.9208, which was significantly higher than for CSF levels of p‐tau181 (CSF‐T: AUC 0.7782) and plasma‐based p‐tau181 (Plasma‐T: AUC 0.7478). Furthermore, analysis of N classification, the AUC for FDG–PET (neuroimaging‐N: AUC 0.9085) was higher than CSF NFL (CSF‐N: AUC 0.7684) and plasma NFL (Plasma‐N: AUC 0.7316). In terms of secondary outcomes analysis that compared participants of MCI versus CN, and MCI versus AD, respectively. The AUCs for CSF‐A (0.6807), neuroimaging‐T (0.6764), and CSF‐N (0.6801) were higher than the same ATN classifications in the MCI versus CN (Figure 1K). However, all the AUC levels were <0.7 in this analysis, suggesting the ATN biomarkers hard to differentiate the MCI from CN. Next, we evaluated the accuracy of ATN biomarkers to identify AD from MCI and found Aβ–PET (neuroimaging‐A: AUC 0.6839), tau‐PET (neuroimaging‐T: AUC 0.7893), and FDG–PET (neuroimaging‐N: AUC 0.8177) demonstrated a significantly higher AUC compared with other ATN classifications (Figure 1L). Finally, ranking results of the A/T/N biomarker features based on their AUC values In different comparisons (AD vs. CN, MCI vs. CN, AD vs. MCI) were shown in Tables S3 and S4, and related cutoff values were shown in Tables S5.

### 3.3 Associations of CSF, plasma, and neuroimaging ATN biomarkers with cognitive function measures

**FIGURE 2:**
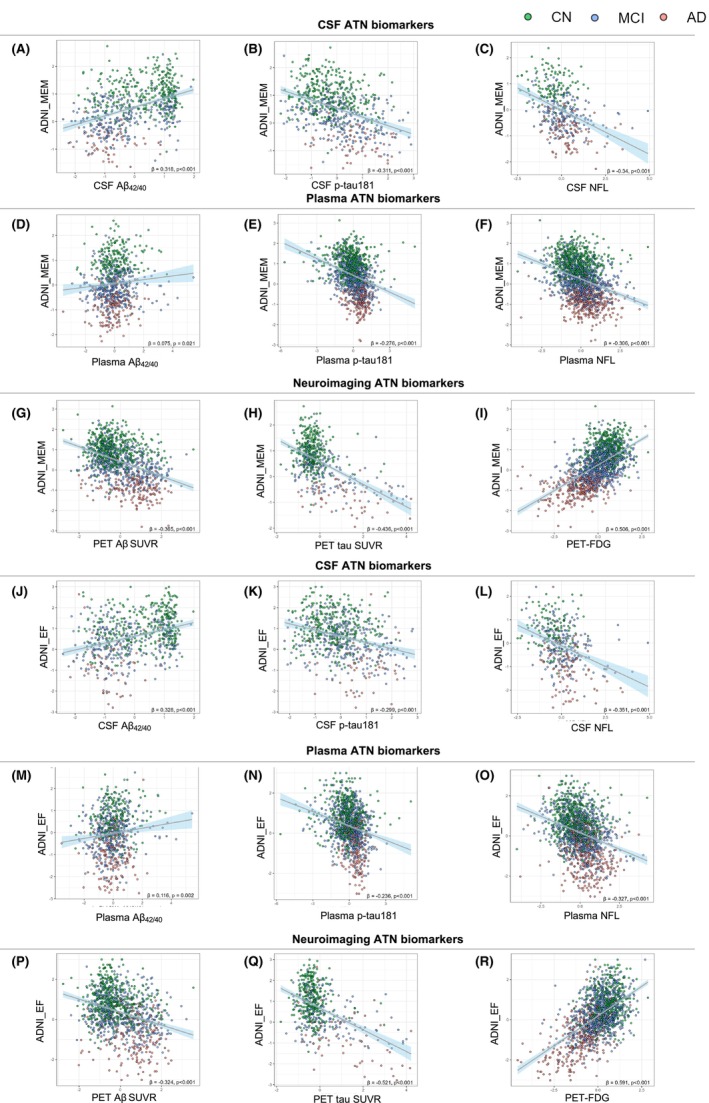
Linear regression models of ATN biomarkers with baseline cognitive scores. All the biomarker levels were natural log transformed and standardized due to non‐normal distribution. Scatter plots showing the correlations of CSF, plasma, and neuroimaging ATN biomarkers with ADNI_MEM (A–I) and ADNI_EF (J–R). Regression coefficient *β* and related *p*‐value were calculated in each linear regression model.

As a result of, age, gender, and education are risk factors for pathological biomarker changes, we adjusted these features by using them as covariates in future linear regression analysis. To test whether the different derived ATN biomarkers in classifying the three subject group pairs are associated with performance on cognitive functioning tests, including ADNI–MEM, and ADNI–EF. Hence, we analyzed each ATN feature's correlation with cognitive functions. All the biomarker levels were natural log transformed and standardized due to non‐normal distribution. We found ADNI_MEM was strongest correlated with Aβ–PET (*β =* −0.365) compared to CSF Aβ (*β =* 0.318) and plasma Aβ (*β =* 0.075) in the “A” classification (Figure 2A,D,G). Of note, tau‐PET (*β =* −0.436) and FDG–PET (*β =* 0.506) were also highly correlated with ADNI–MEM in the “T” and “N” elements, respectively. When linear regression analysis of ADNI–EF in all three groups, cognitive performance was highly associated with the neuroimaging ATN markers, such as Aβ–PET, tau‐PET, and FDG–PET, showing larger coefficients (*β* values) than CSF and plasma ATN biomarkers (Figure 2J–R) except CSF Aβ (*β =* 0.328) was a little higher than Aβ–PET (*β =* 0.324). Moreover, we then analyzed the correlation of the CSF, plasma, and neuroimaging ATN biomarkers in the CN, MCI, and AD groups using heat maps. Correlation coefficients were obtained by the Spearman correlation tests. It showed that the neuroimaging and CSF ATN features were highly correlated with cognitive scales rather than plasma ATN biomarkers in the CN group (Figure S2a, Table S7), whereas the plasma ATN biomarkers started to display a significant effect in the MCI stage (Figure S2b, Table S8). Notably, however, FDG–PET SUVR was more highly correlated with memory than other A/T/N markers in the MCI or AD dementia participants (Figure S2b,c, Tables S8 and S9). Of interest, a similar correlation analysis pattern was represented when combining all subjects, CN&AD, CN&MCI, and MCI&AD (Figure S2d–g, Tables S6, S10–S12), with the correlation values being even larger for the neuroimaging ATN measurements than CSF and plasma‐based ATN biomarkers, especially FDG–PET SUVR, indicating that neuroimaging ATN biomarkers become strongly predictive targets of cognitive performance as cognitive decline progresses from CN to AD. Particularly, tau‐PET (neuroimaging‐T) appeared to be an especially important correlation factor of memory function, as it existed the highest correlation coefficient among the three neuroimaging ATN biomarkers in these cognitive correlation analyses.

### 3.4 The clinical prediction of cognitive decline by CSF, plasma, and neuroimaging ATN biomarker

**FIGURE 3:**
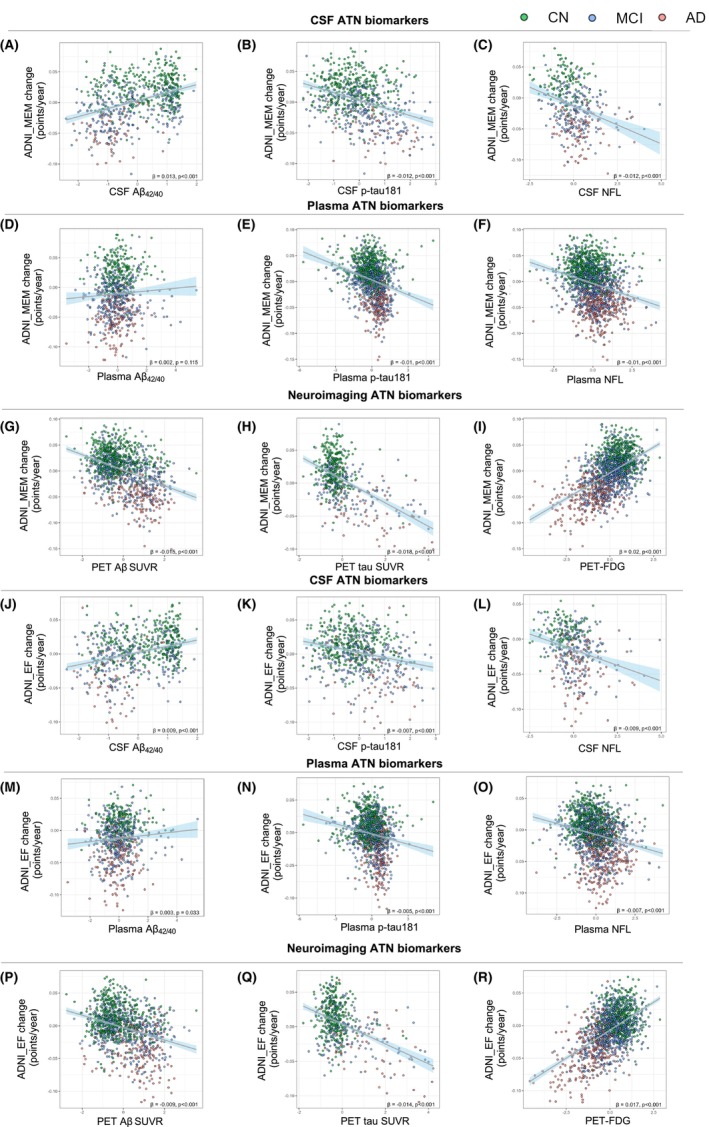
Linear regression models of ATN biomarkers with cognitive annual changes. All the biomarker levels were natural log transformed and standardized due to non‐normal distribution. Cognitive annual changes were got through linear mixed models with 4‐year follow‐up data. LMM had cognitive test scores as the dependent variable and included the independent variables' time (years between baseline and follow‐up time points) for fixed effects and random effects, adjusted for sex, age, and years of education. Scatter plots showing the correlations of CSF, plasma, and neuroimaging ATN biomarkers with ADNI_MEM annual change (A–I) and ADNI–EF annual change (J–R). Regression coefficient *β* and related *p*‐value were calculated in each linear regression model.

To understand if CSF, plasma, and neuroimaging ATN biomarkers are sufficient to predict cognitive decline in CN, MCI, and AD groups, we performed linear mixed‐effect models (LMMs) analysis adjusted for age, gender, and education to get cognitive annual changes. Linear regression analysis showed that Aβ‐PET (*β =* −0.015, *p* < 0.001, Figure 3G), as a neuroimaging‐A classification, had a faster cognitive decline in terms of ADNI–MEM annual changes over 48 months in comparison to CSF Aβ_42/40_ (*β =* 0.013, *p* < 0.001, Figure 3A) and plasma Aβ_42/40_ (*β =* 0.002, *p* = 0.115, Figure 3D). Similarly, faster cognitive decline in tau‐PET (*β =* −0.018, *p* < 0.001, Figure 3H; CSF p‐tau: *β* = −0.012, *p* < 0.001, Figure 3B; Plasma p‐tau: *β* = −0.010, *p* < 0.001, Figure 3E) and FDG–PET uptake (*β* = 0.020, *p* < 0.001, Figure 3I; CSF NFL: *β* = −0.012, *p* < 0.001, Figure 3C; Plasma NFL: *β* = −0.010, *p* < 0.001, Figure 3E) was observed in “T” and “N” biomarker elements, respectively. In addition, other cognitive scales, such as ADNI–EF, as outcome measures were also briefly investigated (Figure 3J–R). Interestingly, consistent with the correlation analysis results, neuroimaging ATN features showed the fastest ADNI–MEM and ADNI–EF changes compared with CSF and plasma ATN elements. General linear mixed models with random intercepts and slopes were also used to examine the influence of higher/lower ATN biomarkers on cognitive performances and decline over time. It showed that “N+” participants, especially “CSF‐N+”, turn out to experience the fastest cognitive decline in each cognitive domain (Detailed information can be found in Table S13). To test the influence of Aβ status on subjects’ cognitive performance in different stages. We then defined neuroimaging‐A+ as pathological positivity of Aβ (Aβ+) and neuroimaging‐A‐ as pathological negativity of Aβ (Aβ−). Baseline cognitive scores and cognitive annual changes were compared between subjects in three diagnostic groups with different Aβ conditions. It showed that both CN and AD subjects had no difference in baseline cognitive scores and cognitive annual changes (Figure S3a–f). It seemed that Aβ status could only make a significant difference in cognitive scores in MCI subjects. In ADNI–EF annual change scores, no significant difference between different Aβ forms could be found in the three diagnostic groups (Figure S3f).

### 3.5 The function of plasma p‐tau 181 in the discrimination of Aβ status of subjects in different diagnostic groups and predicting their 4‐year cognitive trajectory

Roc analysis indicated that plasma p‐tau 181 failed to discriminate Aβ+/Aβ− in CN subjects (Figure S4a). The AUC value turned out to be higher in MCI subjects (AUC: 0.634) and subjects with AD (0.779) (Figure S4b,c). To understand if plasma p‐tau 181 is sufficient to predict cognitive decline in CN and MCI groups, we further performed the linear regression in different Aβ conditions. It showed that plasma p‐tau 181 was not correlated with baseline cognitive scores or cognitive annual changes in the CN group, either Aβ+ or Aβ− (Figure S4d–g). In MCI subjects, plasma p‐tau 181 had a significant correlation with both baseline cognitive scores and cognitive annual changes only in Aβ+ status (Figure S4h–k).

### 3.6 Associations of CSF Aβ_42/40_ with regional tau‐PET and FDG–PET across the AD clinical spectrum

**FIGURE 4:**
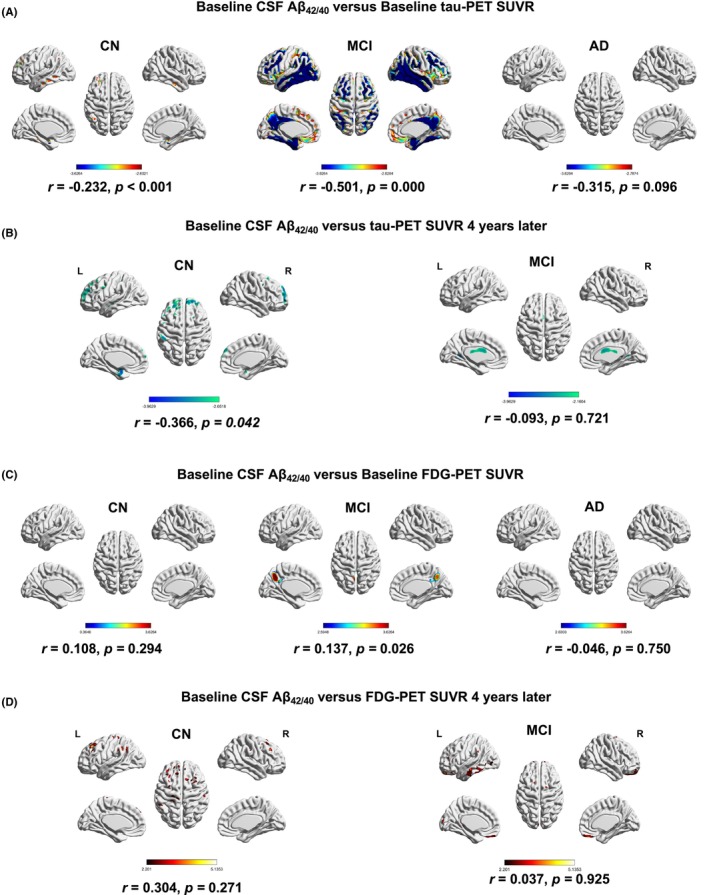
Regional and global associations of CSF Aβ_42/40_ level with baseline and 4‐year‐later cerebral tau pathology and glycometabolism. (A) CSF Aβ_42/40_ level versus baseline tau‐PET SUVR using Voxel‐wise analyses (adjusted for age and sex et al.), (B) CSF Aβ_42/40_ level versus 4‐year‐later tau‐PET SUVR, (C) CSF Aβ_42/40_ level versus baseline FDG–PET SUVR, (D) CSF Aβ_42/40_ level versus 4‐year‐later FDG–PET SUVR. Significant associations in voxel‐wise analyses were determined based on an FWE‐corrected threshold of *p* < 0.05 at the cluster level. Color panels on the bottom display spearman correlation coefficients (*r*).

CSF Aβ_42/40_ (CSF‐A), tau‐PET (neuroimaging‐T), and FDG–PET (neuroimaging‐N) were ranked the top three candidates in diagnosis accuracy for AD versus CN, as well as the high correlation with cognitive performances. To further evaluate the longitudinal relationship between CSF Aβ_42/40_ and tau‐PET and FDG–PET biomarkers. Regarding the tau‐PET domain, we assessed the cross‐sectional associations of CSF Aβ_42/40_ with global tau‐PET SUVR across the AD continuum using voxel‐wise analyses (adjusted for age, sex, and APOE4). Baseline levels of CSF Aβ_42/40_ related with tau accumulation more strongly in subjects with MCI (*r* = −0.501, *p* = 0.000) and CN (*r* = −0.232, *p* < 0.001), while the association was markedly weaker among AD participants (*r* = −0.315, *p* = 0.096, Figure 4A). We then investigated the correlations of baseline CSF Aβ_42/40_ versus longitudinal tau‐PET SUVR 4 years later and found a significant correlation only in the CN group (*r* = −0.366, *p* = 0.042), indicating baseline CSF Aβ_42/40_ change was mildly associated with longitudinal tau accumulation, only marginal and statistically non‐significant associations in MCI subjects (*r* = −0.093, *p* = 0.721, Figure 4B). Moreover, voxel‐wise analyses assessed associations between CSF Aβ_42/40_ and FDG–PET. Like tau‐PET, baseline CSF Aβ_42/40_ demonstrated a mildly significant relationship with FDG–PET SUVR in subjects with MCI (*r* = 0.137, *p* = 0.026), while no significant association found among CN (*r* = 0.108, *p* = 0.294) and AD subjects (*r* = −0.046, *p* = 0.750, Figure 4C). Of note, we investigated whether baseline CSF Aβ_42/40_ correlated with the severity of FDG–PET SUVR 4 years later, and no correlation was found both in the CN (*r* = 0.304, *p* = 0.271) and MCI groups (*r* = 0.037, *p* = 0.925, Figure 4D).

### 3.7 A combination model of the top three ATN biomarkers diagnoses AD

**FIGURE 5:**
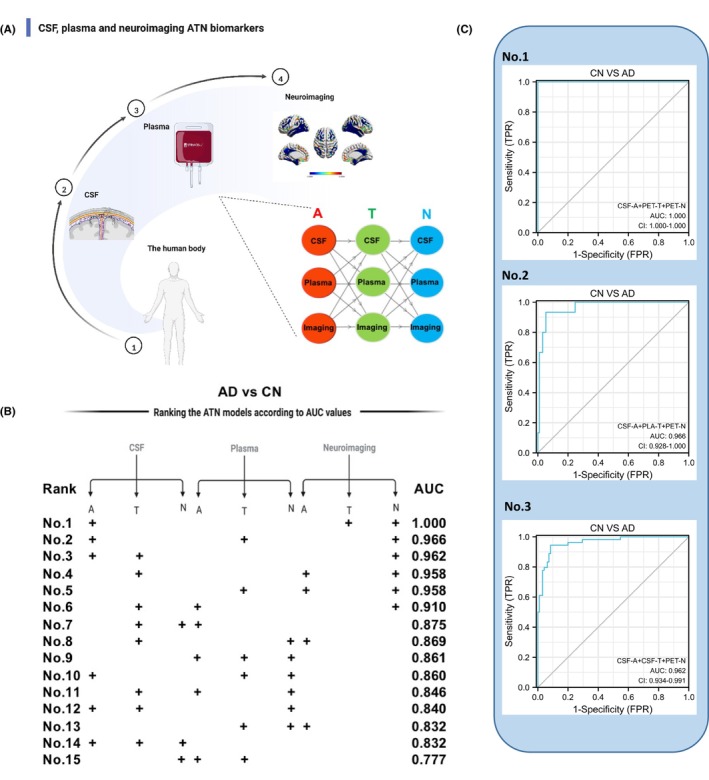
A combination model of the top three ATN biomarkers diagnose AD. We arranged the “A”, “T”, and “N” components to form a serial multivariate logistic model and ranked the ATN biomarker features according to their diagnostic value in this model (A, B). The accuracy of the top three ATN biomarkers distinguishing AD from CN groups is evidenced by AUCs, as shown in (C). The best diagnosis model used in AD versus CN included CSF‐A (CSF Aβ_42/40_), neuroimaging‐T (tau‐PET), and neuroimaging‐N (FDG–PET) with an accuracy of 1.000, followed by neuroimaging‐A (Aβ‐PET), Plasma‐T (plasma p‐tau181) and neuroimaging‐N (FDG–PET) with an accuracy of 0.966, and CAF‐A (CSF Aβ_42/40_), CSF‐T (CSF p‐tau181) and neuroimaging‐N (FDG–PET) showed a similarly high AUC 0.962 (C). The best diagnosis models discriminated MCI versus CN, and MCI versus AD were displayed in Figure S5.

We next sought to assess whether combining CSF, plasma, and neuroimaging ATN biomarkers could further improve the accuracy of diagnosis of AD. We arranged the “A”, “T”, and “N” components to form a serial new diagnosis model and ranked the ATN biomarker features according to their relative importance in each model (Figure 5A,B). The best diagnosis model used in AD versus CN included CSF‐A (CSF Aβ_42/40_), neuroimaging‐T (tau‐PET), and neuroimaging‐N (FDG–PET) with an accuracy of 1.000, followed by neuroimaging‐A (Aβ‐PET), Plasma‐T (plasma p‐tau181) and neuroimaging‐N (FDG–PET) with an accuracy of 0.966, and CAF‐A (CSF Aβ_42/40_), CSF‐T (CSF p‐tau181) and neuroimaging‐N (FDG–PET) showed a similarly high AUC 0.962 (Figure 5C). Of interest, the best diagnosis models discriminated MCI versus CN, and MCI versus AD were displayed in Figure S5. We take these results seriously due to the limited sample sizes, especially the No. 1 model.

## 4 DISCUSSION

This is the first study to our knowledge of full‐scale analysis from all arms of the A/T/N framework, and the core findings of the prospective longitudinal study were as follows: (1) In terms of diagnostic accuracy for AD, the AUCs of CSF Aβ_42/40_ (CSF‐A), tau‐PET (neuroimaging‐T) and FDG–PET (neuroimaging‐N) were ranked as the top three ATN candidates in diagnosis accuracy for AD. (2) Neuroimaging ATN biomarkers demonstrated more strongly predictive targets of cognitive performances in terms of cognitive decline than CSF and plasma ATN categories. Among them, neuroimaging‐T and neuroimaging‐N appeared to be an especially important correlation factors of cognitive function. (3) The status of Aβ might not be effective enough to predict the cognitive decline in subjects with CN or AD, but in subjects with MCI Aβ status could influence a lot. (4) Voxel‐wise analyzed CSF‐A related with tau accumulation and FDG–PET indexes more strongly in subjects with MCI stage. (5) The best‐combined diagnosis model discriminated AD from cognitively unimpaired participants, including CSF‐A, neuroimaging‐T, and Neuroimaging‐N, with an accuracy of 1.000. We provide a detailed description and comprehensive analysis picture of the different sources of ATN biomarkers in the AD continuum.

In this study, we started with the clinical diagnosis and then tested the levels of the CSF, plasma, and neuroimaging ATN biomarkers separately in each diagnostic group (CN, MCI, and AD). The results demonstrated that CSF‐A, neuroimaging‐T, and Neuroimaging‐N, within the same ATN category, might provide better discriminative accuracy for AD. Due to the ATN profiling's lack of interchangeability, such as between CSF and neuroimaging modalities. Hence, capturing the best biomarkers among the ATN systems is increasingly important. Taken together, these results suggest that CSF and Neuroimaging‐derived biomarkers, which are intimately related to the main pathologies, are much more sensitive and accurate than plasma‐derived for AD diagnosis. However, their utility in the clinic is limited in part by their high price and poor accessibility, and plasma‐based biomarkers will likely be a potential paramount prospect in the AD field.^11^ Hence, ongoing research on plasma‐based A/T/N framework biomarkers should be a part of future attempts to close the gaps. Interestingly, several studies based on BioFINDER^41^ and a recent meta‐analysis^42^ have confirmed the high accuracy of plasma p‐tau in diagnosing AD compared to CSF biomarkers. Nevertheless, in the present study, plasma ATN biomarkers were not sufficiently sensitive biomarkers to differentiate AD and predictive cognitive performances. One possible explanation, according to method vision, is that mass spectrometry may not be sensitive enough to measure the plasma biomarkers compared to Simoa assays.

We investigated the associations between ATN biomarkers with cognitive function measures and found neuroimaging ATN biomarkers become more closely predictive of cognitive decline value than other ATN biomarkers, questioning the prognostic cost of CSF and plasma ATN biomarkers, especially the presence of pathological neuroimaging‐T (tau‐PET) and Neuroimaging‐N (FDG–PET) in the brain, which appeared to be a super biomarker with a higher *r* correlation index,^43^ which can be used to future cognitive prediction. Moreover, several pieces of evidence point out a high correlation between tau levels and cognitive deterioration across the entire AD spectrum.^44^ To our knowledge, there are a couple of plausible arguments to make clear why tau‐PET is a vital target in predicting progressive cognitive decline in the clinical AD continuum. In line with other reports, a rapid steeper decline in cognition longitudinally was foreseen by tau‐PET (+) rather than Aβ–PET (+), partially resulting from tau (+) was often coupled with Aβ (+) but not vice versa.^45^ Aβ deposition is the original trigger of tau pathology in the AD continuum, while tau is the concrete driver of neurodegeneration and cognitive decline.^46^ Furthermore, tau diffuses in a relatively stereotypical pattern that is tightly related to clinical status is also one of the potential reasons.

FDG–PET can effectively assess the level of cortical metabolism in the brain area and is also an important indicator reflecting the neurodegeneration dimension of patients with AD.^47^ Studies have shown that in the development course of AD, the abnormal Aβ and tau proteins begin at the early stage of AD onset, while the abnormal glucose uptake and utilization mainly occur in the middle and later stages of the disease.^48^ There are several plausible reasons why hypoglycemia is an important biomarker for predicting progressive cognitive decline in clinical AD. For example, impaired glucose metabolism in the brain is associated with insulin resistance, which in turn exacerbates Aβ deposition. Stanley et al. showed that one of the characteristics of AD was a damaged insulin signal in the brain and abnormal insulin levels in plasma and CSF.^49^

Alternatively, voxel‐wise analyzed CSF‐A related to tau accumulation and FDG–PET more strongly in subjects with MCI stage than CN and AD. The lack of correlation of cognition with Aβ outside MCI is reasonable, given floor and ceiling effects in CN and AD. Furthermore, baseline CSF‐A, more pronounced, was associated with PET‐measured tau aggregation 4 years later in participants with CN (*p* = 0.042), and no‐significant correlation was found in terms of neurodegeneration in CN (*p* = 0.271) and MCI groups (*p* = 0.925). To our knowledge, elevated Aβ is necessary for tau accumulation in the AD continuum. Consistent with this finding, several studies reported that once individuals with elevated Aβ and cognitive impairment, such as the MCI stage, the speed of tau accumulation is up to 2‐fold higher.^50^, ^51^ Interestingly, David et al. demonstrated that participants with elevated Aβ could lead to tau accumulation in the context of high‐Aβ levels (>68 centiloid).^52^ Notably, across the disease progression, given that ATN biomarkers have differing importance in predicting clinical dementia capacity. Tyler et al. reported that Aβ has higher importance in predicting early cognitive impairment (CN and MCI) but may not be sufficient to lead to clinical AD, and glucose uptake has a higher role in the later stage.^53^

We employed AD biomarkers from all arms of the A/T/N framework (CSF, plasma, and Neuroimaging) in a random analysis mode to generate the best optimal cross‐tissue model to accurately diagnose AD and to rank models in order of their importance in the diagnosis accuracy (AUC values). The best‐combined diagnosis model discriminated AD from cognitively unimpaired participants, including CSF‐A, neuroimaging‐T, and Neuroimaging‐N, with an accuracy of 1.000 (The real AUC value is close to 1 based on the limited sample size). Since not all subjects with AD in ADNI were required to be Aβ positive, it is hard to reach the conclusion that an algorithm including A has an accuracy of 1.00 to separate AD and CN. The remarkable thing that should be noted is that PET will have limited acceptance in clinical practice, and performing multiple PET scans for T and N in one patient is quite unlikely to become clinical routine. One shortcoming of this project was that only FDG–PET was considered for N as a neuroimaging marker rather than a brain MRI. Indeed, the disadvantage of this multimodal diagnosis model, including Aβ and tau PET scans and CSF biomarkers, can be complicated and challenging to acquire in clinical settings. Moreover, our findings on CSF‐ Aβ, tau‐PET, or FDG–PET as a single, AD‐specific diagnosis biomarker are enough to differentiate AD from CN. The other limitations of this study including the follow‐up was relatively short (4 years). Notably, the major concern about this project is the lack of an external cohort to validate these findings. Moreover, there are many comparisons made to develop the classification models, which would require reiterating findings and reproducing them in unseen data sets.

Despite the participants from the ADNI cohort being huge, we restricted the patient inclusion to those we had results for CSF, plasma, and neuroimaging ATN biomarkers, which limited the sample size of the results and required validation in other cohorts, which may prove useful for the future prediction and diagnosis of AD. In addition, 18F‐Flortaucipir (tau‐PET) displays considerable off‐target binding in the hippocampus, basal ganglia, or other unspecific binding regions, which may confuse the assessment of tau pathology.^54^

## 5 CONCLUSIONS

Our detailed analysis of the ADNI data enables us to suggest that CSF, plasma, and neuroimaging biomarkers differ considerably within the ATN framework; among these biomarkers, CSF‐A, neuroimaging‐T, and neuroimaging‐N are likely correlating well with AD clinical diagnosis. Moreover, our findings suggest tau‐PET and FDG–PET as reliable biomarkers for predicting cognitive decline.

## AUTHOR CONTRIBUTIONS

HJH and SJQ analyzed the data. XX and QQY searched the literature, XMZ, FFF, and STZ made substantial contributions to conception and replenished the required data. CLX was involved in drafting the manuscript. LS explained the data, EFF helped to revise the paper.

## FUNDING INFORMATION

Supported by the Projects of the National Science Foundation of China (Nos. 81600977 and 82271469) and the Projects of the Natural Science Foundation of Zhejiang Province (Y19H090059 and LZ23H090001).

## CONFLICT OF INTEREST STATEMENT

No commercial or financial relationships could be construed as a potential conflict of interest.

## Supporting information


Appendix S1

## Data Availability

ADNI has made publicly available the data used in this study in the Neuroimaging Laboratory (LONI) database.
