# Supplementary material for: Alzheimer's disease diagnostic accuracy by fluid and neuroimaging ATN framework
Source: CNS Neurosci Ther. 2023 Jul 12;30(2):e14357. doi: 10.1111/cns.14357 (PMC10848089; doi:10.1111/cns.14357)
Supplement: Supplementary file 1 — Appendix S1 [file CNS-30-e14357-s001.pdf]

Supplementary documents

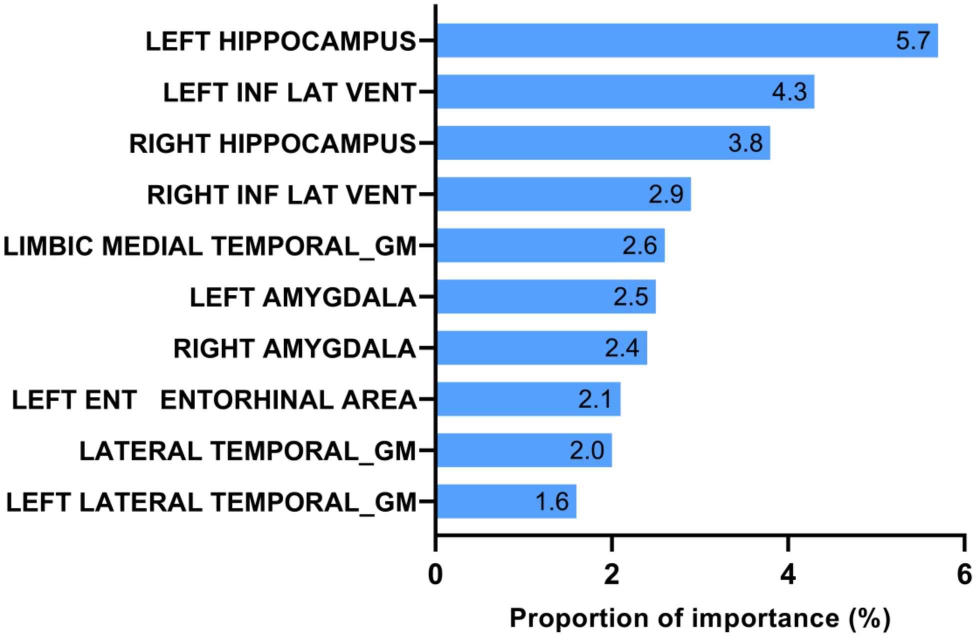

LEFT HIPPOCAMPUS
LEFT INF LAT VENT
RIGHT HIPPOCAMPUS
RIGHT INF LAT VENT
LIMBIC MEDIAL TEMPORAL_GM
LEFT AMYGDALA
RIGHT AMYGDALA
LEFT ENT ENTORHINAL AREA
LATERAL TEMPORAL_GM
LEFT LATERAL TEMPORAL_GM
5.7
4.3
3.8
2.9
2.6
2.5
2.4
2.1
2.0
1.6
0
2
4
6
Proportion of importance (%)

Supple Fig. 1

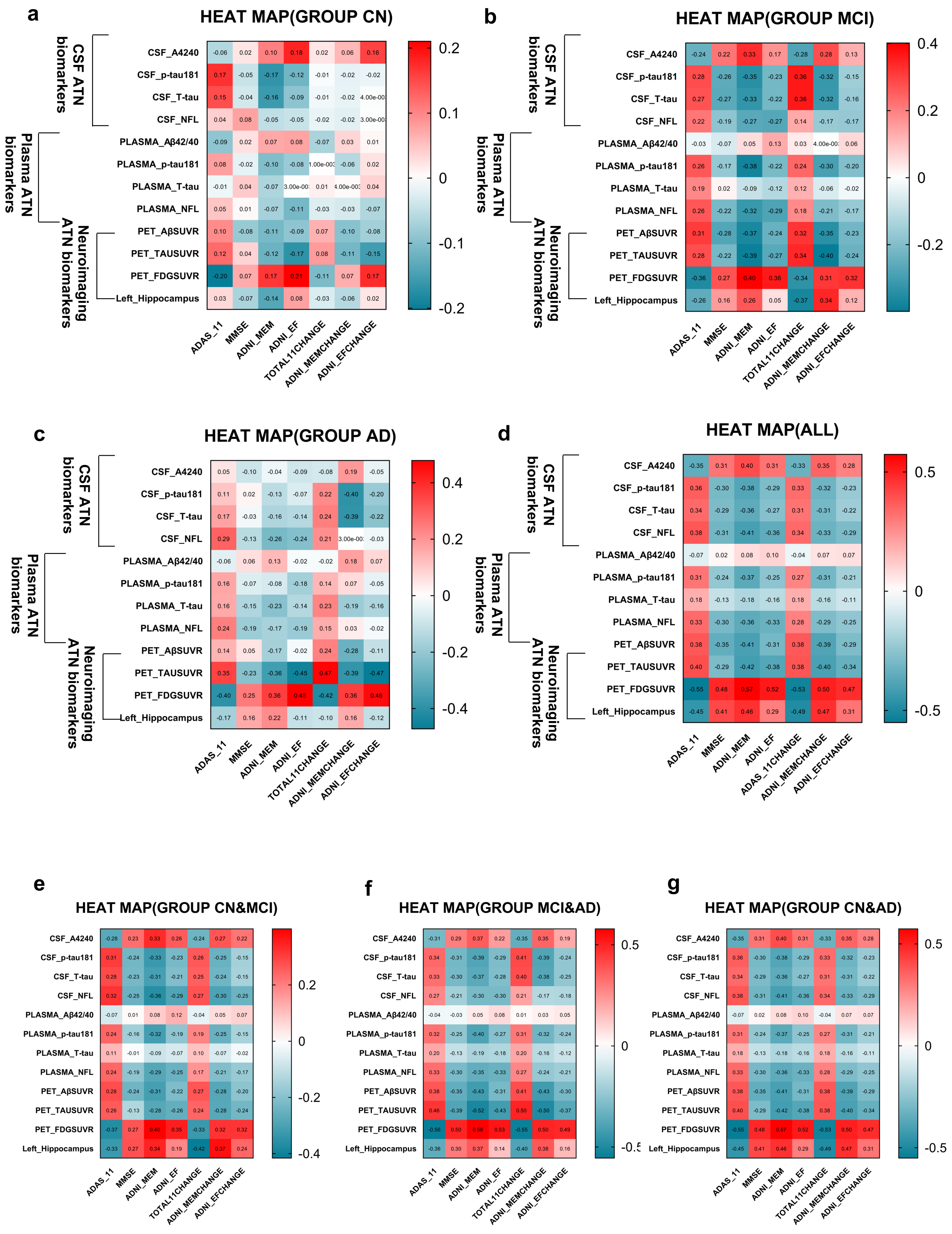

Supple Fig. 2

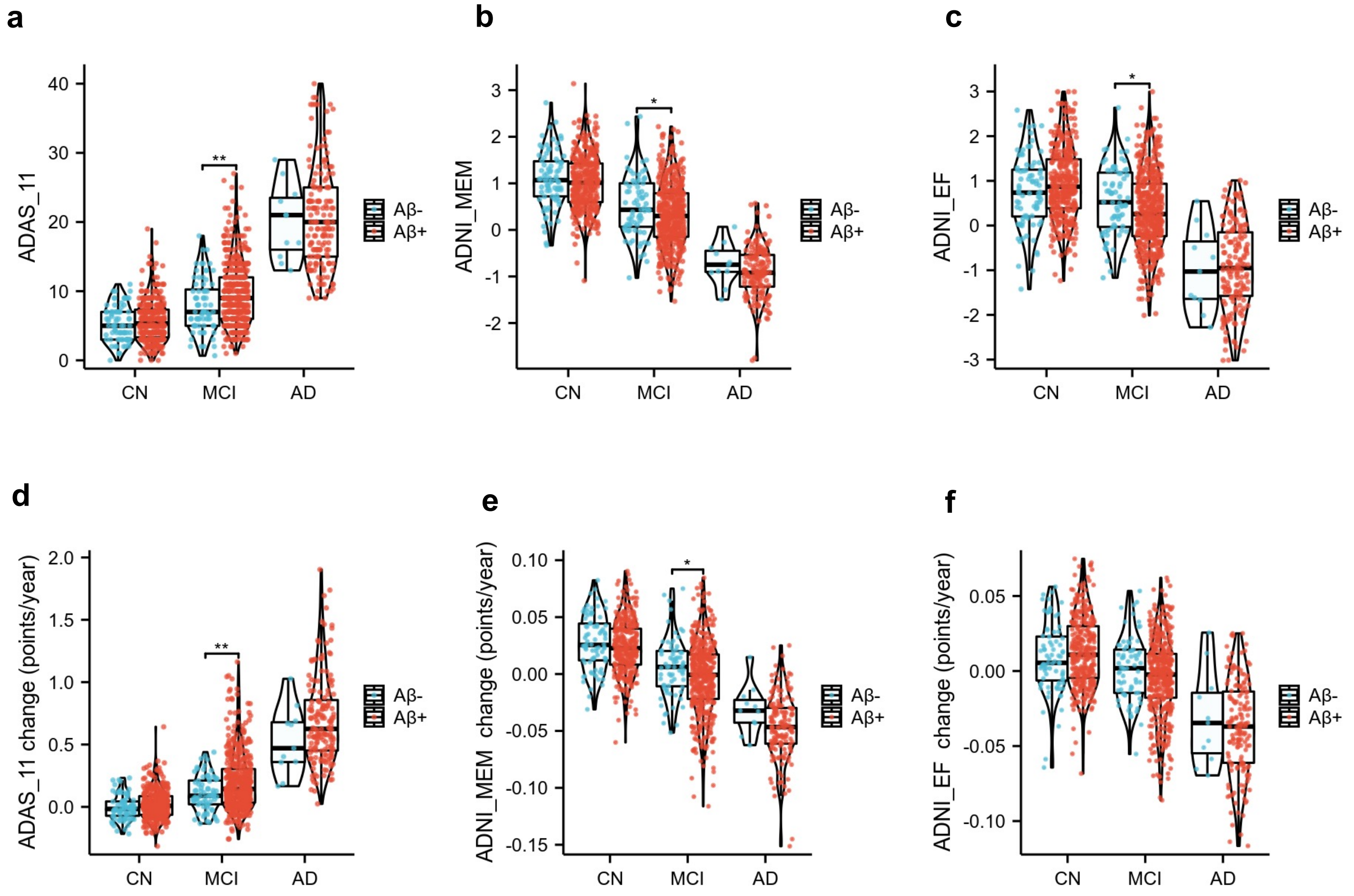
a
ADAS_11
40
30
20
10
0
**
CN
MCI
AD
Aβ-
Aβ+
b
ADNI_MEM
3
2
1
0
-1
-2
*
CN
MCI
AD
Aβ-
Aβ+
c
ADNI_EF
3
2
1
0
-1
-2
-3
*
CN
MCI
AD
Aβ-
Aβ+
d
ADAS_11 change (points/year)
2.0
1.5
1.0
0.5
0.0
**
CN
MCI
AD
Aβ-
Aβ+
e
ADNI_MEM change (points/year)
0.10
0.05
0.00
-0.05
-0.10
-0.15
*
CN
MCI
AD
Aβ-
Aβ+
f
ADNI_EF change (points/year)
0.05
0.00
-0.05
-0.10
CN
MCI
AD
Aβ-
Aβ+

Supple Fig. 3

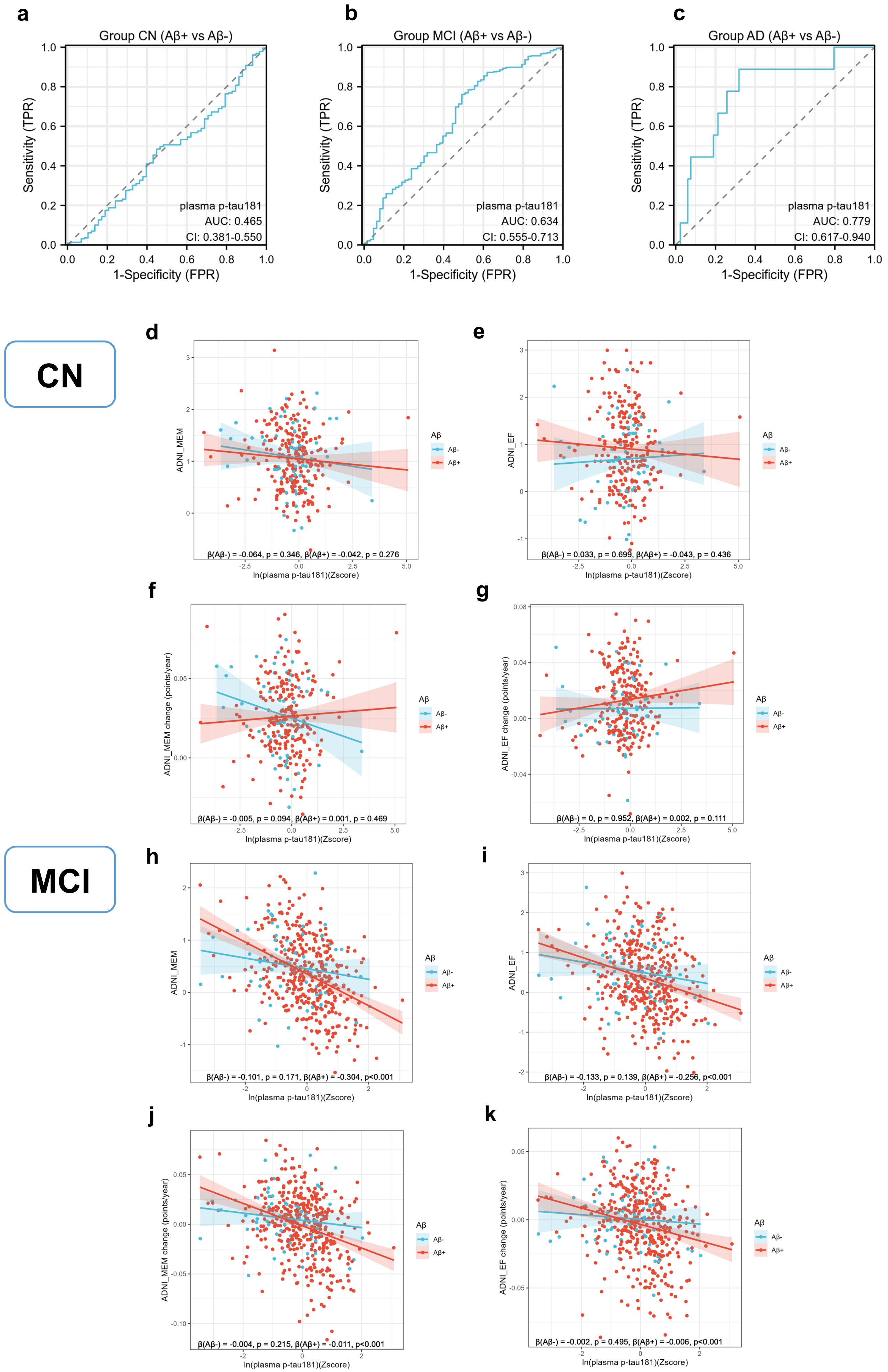
a
Group CN (Aβ+ vs Aβ-)
Sensitivity (TPR)
1-Specificity (FPR)
plasma p-tau181
AUC: 0.465
CI: 0.381-0.550
b
Group MCI (Aβ+ vs Aβ-)
Sensitivity (TPR)
1-Specificity (FPR)
plasma p-tau181
AUC: 0.634
CI: 0.555-0.713
c
Group AD (Aβ+ vs Aβ-)
Sensitivity (TPR)
1-Specificity (FPR)
plasma p-tau181
AUC: 0.779
CI: 0.617-0.940
CN
d
ADNI_MEM
β(Aβ-) = -0.064, p = 0.346, β(Aβ+) = -0.042, p = 0.276
ln(plasma p-tau181)(Zscore)
Aβ
Aβ-
Aβ+
e
ADNI_EF
β(Aβ-) = 0.033, p = 0.699, β(Aβ+) = -0.043, p = 0.436
ln(plasma p-tau181)(Zscore)
f
ADNI_MEM change (points/year)
β(Aβ-) = -0.005, p = 0.094, β(Aβ+) = 0.001, p = 0.469
ln(plasma p-tau181)(Zscore)
g
ADNI_EF change (points/year)
β(Aβ-) = 0, p = 0.952, β(Aβ+) = 0.002, p = 0.111
ln(plasma p-tau181)(Zscore)
MCI
h
ADNI_MEM
β(Aβ-) = -0.101, p = 0.171, β(Aβ+) = -0.304, p<0.001
ln(plasma p-tau181)(Zscore)
i
ADNI_EF
β(Aβ-) = -0.133, p = 0.139, β(Aβ+) = -0.256, p<0.001
ln(plasma p-tau181)(Zscore)
j
ADNI_MEM change (points/year)
β(Aβ-) = -0.004, p = 0.215, β(Aβ+) = -0.011, p<0.001
ln(plasma p-tau181)(Zscore)
k
ADNI_EF change (points/year)
β(Aβ-) = -0.002, p = 0.495, β(Aβ+) = -0.006, p<0.001
ln(plasma p-tau181)(Zscore)

Supple Fig. 4

a

MCI vs CN

Ranking the ATN models according to AUC values

Rank	CSF A	CSF T	CSF N	Plasma A	Plasma T	Plasma N	Neuroimaging A	Neuroimaging T	Neuroimaging N	AUC
No.1					+		+		+	0.912
No.2	+							+	+	0.793
No.3	+	+	+							0.769
No.4		+		+					+	0.744
No.5		+	+	+						0.718
No.6	+				+				+	0.708
No.7	+		+		+					0.707
No.8	+	+							+	0.698
No.9		+					+		+	0.697
No.10				+	+				+	0.672
No.11			+	+	+					0.669
No.12				+	+	+				0.657
No.13	+				+	+				0.655
No.14	+	+				+				0.648
No.15		+				+	+			0.627
No.16					+	+	+			0.610
No.17		+		+		+				0.607

c

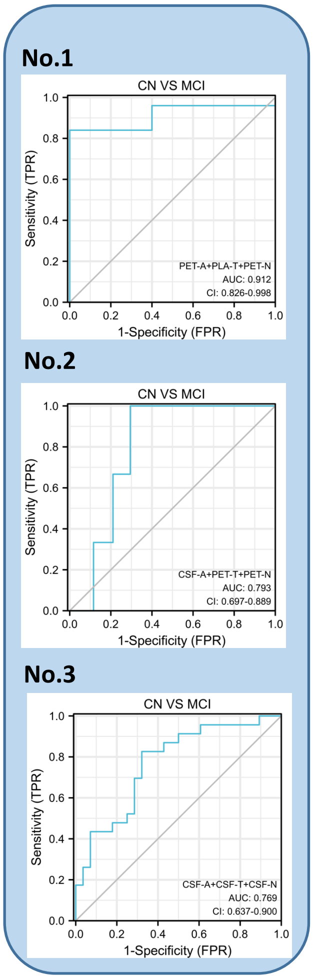

b

MCI vs AD

Ranking the ATN models according to AUC values

Rank	CSF A	CSF T	CSF N	Plasma A	Plasma T	Plasma N	Neuroimaging A	Neuroimaging T	Neuroimaging N	AUC
No.1	+							+	+	0.873
No.2		+					+		+	0.852
No.3	+	+							+	0.845
No.4	+				+				+	0.833
No.5					+		+		+	0.832
No.6			+	+	+					0.790
No.7	+				+	+				0.779
No.8		+		+		+				0.754
No.9	+	+				+				0.753
No.10					+	+	+			0.750
No.11		+				+	+			0.746
No.12		+		+					+	0.730
No.13				+	+	+				0.730
No.14		+	+	+						0.727
No.15	+	+	+							0.674

d

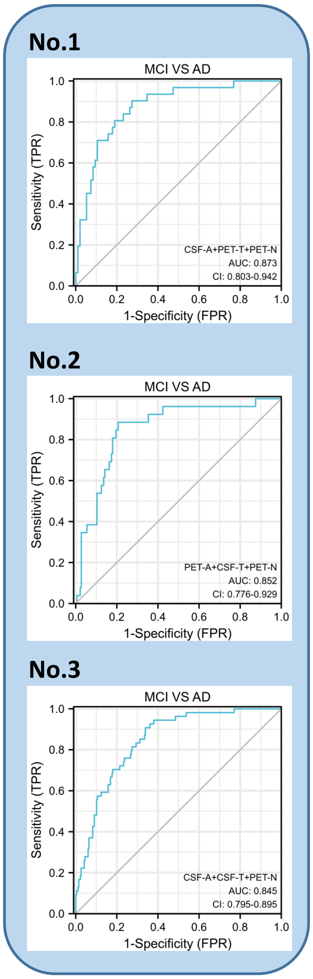

Supple Fig. 5

Supplementary Table 1: Comparison of AUC values of CN/AD for biomarkers in the same dimension

AT(N) biomarkers	CN (N)	AD (N)	CN/AD AUC [95%CI]	p value vs 2	p value vs 3	p value vs 4
**CSF ATN biomarkers**						
1.CSF Aβ42/40	312	58	0.838 [0.792, 0.884]	0.152	0.076	0.079
2.CSF p-Tau	311	58	0.778 [0.710, 0.847]		0.762	0.833
3.CSF T-tau	311	58	0.763 [0.694, 0.832]			0.917
4.CSF NfL	111	95	0.768 [0.705, 0.832]			
**Plasma ATN biomarkers**						
1.Plasma Aβ42/40	212	164	0.537 [0.479, 0.596]	<0.001	0.132	<0.001
2.Plasma p-Tau181	428	147	0.748 [0.703, 0.792]		<0.001	0.578
3.Plasma T-tau	194	188	0.599 [0.543, 0.656]			<0.001
4.Plasma NfL	508	331	0.732 [0.698, 0.766]			
**Neuroimaging ATN biomarkers**						
1.PET SUVR(Aβ)	422	171	0.791 [0.748, 0.834]	0.038	<0.001	0.043
2.PET SUVR(Tau)	300	61	0.871 [0.809, 0.933]		0.258	0.593
3.PET SUVR(FDG)	402	295	0.909 [0.887, 0.930]			0.011
4.Volume of Left Hippocampus	228	191	0.851 [0.812, 0.889]			

The AUC results of area under ROC curve were expressed as AUC value [95% confidence interval]. Delong test was used to calculate the 95% confidence interval and the AUC values of different biomarkers were compared in CN vs AD.

Supplementary Table 2: New AT(N) frame biomarkers

ATN biomarkers	CSF	Plasma	Neuroimaging
**A classification**	CSF Aβ42/40	Plasma Aβ42/40	PET SUVR(Aβ)
**T classification**	CSF p-Tau181	Plasma p-Tau181	PET SUVR(Tau)
**N classification**	CSF NfL	Plasma NfL	PET SUVR(FDG)

The best framework biomarkers were selected through the comparison of the AUC values in different comparisons.

Supplementary Table 3: AUC values for AT(N) biomarkers in different comparisons

ATN biomarkers	CN (N)	MCI (N)	AD (N)	AUC [95%CI] (CN vs MCI)	AUC [95%CI] (CN vs AD)	AUC [95%CI] (MCI vs AD)
**CSF ATN biomarkers**						
CSF_A$\beta_{42/40}$	312	288	58	0.657 [0.613, 0.701]	0.838 [0.792, 0.884]	0.681 [0.616, 0.746]
CSF p-tau	311	288	58	0.635 [0.591, 0.680]	0.778 [0.710, 0.847]	0.659 [0.584, 0.734]
CSF T-tau	111	193	95	0.680 [0.619, 0.741]	0.768 [0.705, 0.832]	0.591 [0.523, 0.659]
**Plasma ATN biomarkers**						
Plasma_A$\beta_{42/40}$	212	356	164	0.538 [0.490, 0.587]	0.537 [0.479, 0.596]	0.496 [0.444, 0.548]
Plasma p-tau	428	613	147	0.589 [0.554, 0.623]	0.748 [0.703, 0.792]	0.648 [0.603, 0.693]
Plasma T-tau	508	740	331	0.594 [0.562, 0.626]	0.732 [0.698, 0.766]	0.637 [0.602, 0.671]
**Neuroimaging ATN biomarkers**						
PET SUVR(Aβ)	422	530	171	0.607 [0.572, 0.643]	0.791 [0.748, 0.834]	0.684 [0.639, 0.729]
PET SUVR (Tau)	300	177	61	0.638 [0.583, 0.693]	0.871 [0.809, 0.933]	0.741 [0.668, 0.814]
PET SUVR (FDG)	402	812	295	0.644 [0.612, 0.676]	0.909 [0.887, 0.930]	0.818 [0.790, 0.845]

The AUC result of area under ROC curve was expressed as AUC value [95% confidence interval]. The 95% confidence interval was calculated by Delong test and pairwise comparison was conducted.

Supplementary Table 4: Ranking the A/T/N biomarker features based on their AUC value

	Rank	AD vs CN	MCI vs CN	AD vs MCI
**A classification**	**No. 1**	CSF-A	CSF-A	Neuroimaging-A
	No. 2	Neuroimaging-A	Neuroimaging-A	CSF-A
	No. 3	Plasma-A	Plasma-A	Plasma-A
**T classification**	**No. 1**	Neuroimaging-T	Neuroimaging-T	Neuroimaging-T
	No. 2	CSF-T	CSF-T	CSF-T
	No. 3	Plasma-T	Plasma-T	Plasma-T
**N classification**	**No. 1**	Neuroimaging-N	CSF-N	Neuroimaging-N
	No. 2	CSF-N	Neuroimaging-N	Plasma-N
	No. 3	Plasma-N	Plasma-N	CSF-N

ATN biomarkers are ranked depend on the AUC value in different comparisons (AD vs CN, MCI vs CN, AD vs MCI) and top three biomarkers are presented.

Supplementary Table 5: Cutoff values of AT(N) biomarkers in different comparisons

ATN biomarkers	AD vs CN	MCI vs CN	AD vs MCI
**CSF ATN biomarkers**			
CSF $A\beta_{42/40}$	0.0456	0.0558	0.0467
CSF p-Tau181 (pg/ml)	25.99	21.70	28.54
CSF NfL (pg/ml)	1296	1288	1197
**Plasma ATN biomarkers**			
Plasma $A\beta_{42/40}$	0.2760	0.1959	0.2775
Plasma p-Tau181 (pg/ml)	12.860	18.708	18.392
Plasma NfL (pg/ml)	32.35	30.40	32.30
**Neuroimaging ATN biomarkers**			
PET SUVR(Aβ)	1.4174	1.3918	1.4774
PET SUVR(Tau)	1.2452	1.2686	1.4248
PET SUVR (FDG)	1.1543	1.2443	1.1486

By calculating the Yoden index (sensitivity + specificity -1) of each marker to identify different cognitive groups, and sorting the results, the biomarker level corresponding to the maximum value can be obtained, which is the optimal critical point of the marker, also known as the cutoff value.

Supplementary Table 6: Correlation of CSF, plasma and neuroimaging ATN biomarkers with memory tests

ATN biomarkers (ρ)	ADAS_11	MMSE	ADNI_MEM	ADNI_EF	ADAS_11 change	ADNI_MEM change	ADNI_EF change
**CSF ATN biomarkers**							
CSF_$A\beta_{42/40}$	-0.354*	0.311*	0.398*	0.313*	-0.33*	0.347*	0.281*
CSF p-tau	0.361*	-0.302*	-0.383*	-0.289*	0.329*	-0.321*	-0.228*
CSF T-tau	0.337*	-0.291*	-0.362*	-0.271*	0.314*	-0.307*	-0.217*
CSF_NFL	0.379*	-0.314*	-0.409*	-0.355*	0.336*	-0.328*	-0.291*
**Plasma ATN biomarkers**							
Plasma_$A\beta_{42/40}$	-0.072	0.019	0.075*	0.098*	-0.043	0.065	0.068
Plasma p-tau	0.309*	-0.241*	-0.367*	-0.254*	0.272*	-0.311*	-0.208*
Plasma T-tau	0.18*	-0.13*	-0.179*	-0.156*	0.181*	-0.156*	-0.114*
Plasma_NFL	0.331*	-0.299*	-0.361*	-0.33*	0.281*	-0.287*	-0.248*
**Neuroimaging ATN biomarkers**							
PET SUVR(Aβ)	0.377*	-0.347*	-0.406*	-0.307*	0.375*	-0.39*	-0.289*
PET SUVR (Tau)	0.396*	-0.293*	-0.417*	-0.38*	0.383*	-0.403*	-0.338*
PET SUVR (FDG)	-0.551*	0.482*	0.573*	0.515*	-0.531*	0.503*	0.474*
Left_Hippocampus	-0.452*	0.411*	0.464*	0.288*	-0.492*	0.467*	0.311*

The ρ is the spearman correlation coefficients. Asterisk (*) next to r value indicates statistical significance ($p < 0.05$). NA, not applicable; CSF, cerebro-Spinal Fluid; Aβ, amyloid beta; p-Tau, phosphorylated tau 181; T-Tau, total tau; NfL, neurofilament light; PET, positron emission tomography; SUVR, standardized uptake value ratio; FDG, fluorodeoxyglucose; ADAS, Alzheimer's disease assessment scale-cognitive subscale; MMSE, mini-mental state examination; CDRSB, clinical dementia rating sum of boxes; ADNI-MEM, composite memory score; ADNI-EF, composite executive functioning score.

Supplementary Table 7: Correlation of ATN biomarkers with memory tests in the CN group

ATN biomarkers (ρ)	ADAS-11	MMSE	ADNI-MEM	ADNI-EF	ADAS-11 change	ADNI-MEM change	ADNI-EF change
**CSF ATN biomarkers**							
CSF_A$\beta_{42/40}$	-0.062	0.02	0.104	0.18*	0.02	0.059	0.155*
CSF p-tau	0.17*	-0.045	-0.168*	-0.117*	-0.005	-0.021	-0.017
CSF T-tau	0.147*	-0.037	-0.159*	-0.093	-0.011	-0.019	-0.004
CSF_NFL	0.04	0.076	-0.052	-0.045	-0.018	-0.024	-0.003
**Plasma ATN biomarkers**							
Plasma_A$\beta_{42/40}$	-0.092	0.024	0.071	0.075	-0.07	0.034	0.007
Plasma p-tau	0.079	-0.021	-0.103*	-0.083	0.001	-0.061	0.023
Plasma T-tau	-0.014	0.043	-0.067	-0.003	0.013	0.004	0.036
Plasma_NFL	0.047	0.005	-0.073	-0.112*	-0.028	-0.034	-0.065
**Neuroimaging ATN biomarkers**							
PET SUVR(Aβ)	0.095	-0.084	-0.111*	-0.092	0.065	-0.097*	-0.076
PET SUVR (Tau)	0.122*	0.036	-0.12*	-0.17*	0.082	-0.113	-0.154*
PET SUVR (FDG)	-0.202*	0.069	0.165*	0.21*	-0.107*	0.066	0.173*
Left_Hippocampus	0.031	-0.069	-0.139*	0.077	-0.031	-0.06	0.02

The ρ is the spearman correlation coefficients. Asterisk (*) next to r value indicates statistical significance ($p < 0.05$). NA, not applicable; CSF, cerebro-Spinal Fluid; Aβ, amyloid beta; p-Tau, phosphorylated tau 181; T-Tau, total tau; NfL, neurofilament light; PET, positron emission tomography; SUVR, standardized uptake value ratio; FDG, fluorodeoxyglucose; ADAS, Alzheimer's disease assessment scale-cognitive subscale; MMSE, mini-mental state examination; CDRSB, clinical dementia rating sum of boxes; ADNI-MEM, composite memory score; ADNI-EF, composite executive functioning score. ADAS-11 change, the average every-year decline of ADAS-11 points in 4 years; ADNI-MEM change, the average every-year decline of ADNI-MEM points in 4 years; ADNI-EF change, the average every-year decline of ADNI-EF points in 4 years.

Supplementary Table 8: Correlation of ATN biomarkers with memory tests in the MCI group

ATN biomarkers (ρ)	ADAS-11	MMSE	ADNI-MEM	ADNI-EF	ADAS-11 change	ADNI-MEM change	ADNI-EF change
**CSF ATN biomarkers**							
CSF_A$\beta_{42/40}$	-0.242*	0.217*	0.33*	0.171*	-0.283*	0.277*	0.125*
CSF p-tau	0.281*	-0.262*	-0.347*	-0.225*	0.363*	-0.323*	-0.154*
CSF T-tau	0.271*	-0.265*	-0.329*	-0.224*	0.363*	-0.319*	-0.164*
CSF_NFL	0.215*	-0.185*	-0.268*	-0.274*	0.135	-0.167*	-0.172*
**Plasma ATN biomarkers**							
Plasma_A$\beta_{42/40}$	-0.029	-0.065	0.052	0.131*	0.031	-0.004	0.061
Plasma p-tau	0.263*	-0.173*	-0.382*	-0.216*	0.24*	-0.296*	-0.202*
Plasma T-tau	0.188*	0.018	-0.092	-0.121	0.124	-0.063	-0.024
Plasma_NFL	0.263*	-0.218*	-0.321*	-0.286*	0.183*	-0.209*	-0.169*
**Neuroimaging ATN biomarkers**							
PET SUVR(Aβ)	0.308*	-0.277*	-0.37*	-0.244*	0.319*	-0.345*	-0.226*
PET SUVR (Tau)	0.277*	-0.221*	-0.394*	-0.269*	0.339*	-0.399*	-0.239*
PET SUVR (FDG)	-0.361*	0.27*	0.401*	0.36*	-0.335*	0.313*	0.321*
Left_Hippocampus	-0.257*	0.157*	0.264*	0.048	-0.374*	0.341*	0.119*

The ρ is the spearman correlation coefficients. Asterisk (*) next to r value indicates statistical significance ($p < 0.05$). NA, not applicable; CSF, cerebro-Spinal Fluid;

Aβ, amyloid beta; p-Tau, phosphorylated tau 181; T-Tau, total tau; NfL, neurofilament light; PET, positron emission tomography; SUVR, standardized uptake value ratio; FDG, fluorodeoxyglucose; ADAS, Alzheimer's disease assessment scale-cognitive subscale; MMSE, mini-mental state examination; CDRSB, clinical dementia rating sum of boxes; ADNI-MEM, composite memory score; ADNI-EF, composite executive functioning score. ADAS-11 change, the average every-year decline of ADAS-11 points in 4 years; ADNI-MEM change, the average every-year decline of ADNI-MEM points in 4 years; ADNI-EF change, the average every-year decline of ADNI_EF points in 4 years.

Supplementary Table 9: Correlation of ATN biomarkers with memory tests in the AD dementia participants

ATN biomarkers (ρ)	ADAS-11	MMSE	ADNI-MEM	ADNI-EF	ADAS-11 change	ADNI-MEM change	ADNI-EF change
**CSF ATN biomarkers**							
CSF_A$\beta_{42/40}$	0.05	-0.098	-0.035	-0.093	-0.084	0.193	-0.045
CSF p-tau	0.108	0.02	-0.13	-0.072	0.218	-0.396*	-0.195
CSF T-tau	0.173	-0.026	-0.155	-0.142	0.239	-0.393*	-0.223
CSF_NFL	0.293*	-0.134	-0.256*	-0.235*	0.209*	-0.003	-0.032
**Plasma ATN biomarkers**							
Plasma_A$\beta_{42/40}$	-0.055	0.06	0.13	-0.018	-0.02	0.176*	0.074
Plasma p-tau	0.164*	-0.065	-0.081	-0.176*	0.135	0.067	-0.048
Plasma T-tau	0.16*	-0.154*	-0.225*	-0.143	0.229*	-0.188*	-0.156*
Plasma_NFL	0.239*	-0.189*	-0.166*	-0.194*	0.154*	0.032	-0.022
**Neuroimaging ATN biomarkers**							
PET SUVR(Aβ)	0.14	0.05	-0.172*	-0.021	0.235*	-0.284*	-0.114
PET SUVR (Tau)	0.351*	-0.23	-0.362*	-0.454*	0.469*	-0.391*	-0.472*
PET SUVR (FDG)	-0.401*	0.247*	0.36*	0.478*	-0.423*	0.36*	0.458*
Left_Hippocampus	-0.174*	0.161*	0.215*	-0.111	-0.099	0.157*	-0.118

The ρ is the spearman correlation coefficients. Asterisk (*) next to *r* value indicates statistical significance ($P < 0.05$). NA, not applicable; CSF, cerebro-Spinal Fluid; Aβ, amyloid beta; p-Tau, phosphorylated tau 181; T-Tau, total tau; NfL, neurofilament light; PET, positron emission tomography; SUVR, standardized uptake value ratio; FDG, fluorodeoxyglucose; ADAS, Alzheimer's disease assessment scale-cognitive subscale; MMSE, mini-mental state examination; CDRSB, clinical dementia rating sum of boxes; ADNI-MEM, composite memory score; ADNI-EF, composite executive functioning score. ADAS-11 change, the average every-year decline of ADAS-11 points in 4 years; ADNI-MEM change, the average every-year decline of ADNI-MEM points in 4 years; ADNI-EF change, the average every-year decline of ADNI-EF points in 4 years.

Supplementary Table 10: Correlation of ATN biomarkers with memory tests among CN & AD

ATN biomarkers (ρ)	ADAS-11	MMSE	ADNI-MEM	ADNI-EF	ADAS-11 change	ADNI-MEM change	ADNI-EF change
**CSF ATN biomarkers**							
CSF_A$\beta_{42/40}$	-0.354*	0.311*	0.398*	0.313*	-0.33*	0.347*	0.281*
CSF p-tau	0.361*	-0.302*	-0.383*	-0.289*	0.329*	-0.321*	-0.228*
CSF T-tau	0.337*	-0.291*	-0.362*	-0.271*	0.314*	-0.307*	-0.217*
CSF_NFL	0.379*	-0.314*	-0.409*	-0.355*	0.336*	-0.328*	-0.291*
**Plasma ATN biomarkers**							
Plasma_A$\beta_{42/40}$	-0.072	0.019	0.075*	0.098*	-0.043	0.065	0.068
Plasma p-tau	0.309*	-0.241*	-0.367*	-0.254*	0.272*	-0.311*	-0.208*
Plasma T-tau	0.18*	-0.13*	-0.179*	-0.156*	0.181*	-0.156*	-0.114*
Plasma_NFL	0.331*	-0.299*	-0.361*	-0.33*	0.281*	-0.287*	-0.248*
**Neuroimaging ATN biomarkers**							
PET SUVR(Aβ)	0.377*	-0.347*	-0.406*	-0.307*	0.375*	-0.39*	-0.289*
PET SUVR (Tau)	0.396*	-0.293*	-0.417*	-0.38*	0.383*	-0.403*	-0.338*
PET SUVR (FDG)	-0.551*	0.482*	0.573*	0.515*	-0.531*	0.503*	0.474*
Left_Hippocampus	-0.452*	0.411*	0.464*	0.288*	-0.492*	0.467*	0.311*

The ρ is the spearman correlation coefficients. Asterisk (*) next to r value indicates statistical significance ($p < 0.05$). NA, not applicable; CSF, cerebro-Spinal Fluid; Aβ, amyloid beta; p-Tau, phosphorylated tau 181; T-Tau, total tau; NfL, neurofilament light; PET, positron emission tomography; SUVR, standardized uptake value ratio; FDG, fluorodeoxyglucose; ADAS, Alzheimer's disease assessment scale-cognitive subscale; MMSE, mini-mental state examination; CDRSB, clinical dementia rating sum of boxes; ADNI-MEM, composite memory score; ADNI-EF, composite executive functioning score. ADAS-11 change, the average every-year decline of ADAS-11 points in 4 years; ADNI-MEM change, the average every-year decline of ADNI-MEM points in 4 years; ADNI-EF change, the average every-year decline of ADNI-EF points in 4 years.

Supplementary Table 11: Correlation of ATN biomarkers with memory tests among CN & MCI

ATN biomarkers (ρ)	ADAS-11	MMSE	ADNI-MEM	ADNI-EF	ADAS-11 change	ADNI-MEM change	ADNI-EF change
**CSF ATN biomarkers**							
CSF_A$\beta_{42/40}$	-0.279*	0.226*	0.332*	0.256*	-0.244*	0.266*	0.217*
CSF p-tau	0.306*	-0.236*	-0.33*	-0.227*	0.261*	-0.251*	-0.153*
CSF T-tau	0.283*	-0.23*	-0.311*	-0.213*	0.25*	-0.24*	-0.147*
CSF_NFL	0.318*	-0.251*	-0.364*	-0.294*	0.268*	-0.299*	-0.248*
**Plasma ATN biomarkers**							
Plasma_A$\beta_{42/40}$	-0.073	0.009	0.081	0.122*	-0.042	0.053	0.07
Plasma p-tau	0.24*	-0.158*	-0.315*	-0.19*	0.19*	-0.253*	-0.145*
Plasma T-tau	0.112*	-0.011	-0.09	-0.069	0.095	-0.066	-0.022
Plasma_NFL	0.238*	-0.188*	-0.286*	-0.25*	0.166*	-0.212*	-0.174*
**Neuroimaging ATN biomarkers**							
PET SUVR(Aβ)	0.278*	-0.239*	-0.312*	-0.217*	0.265*	-0.284*	-0.195*
PET SUVR (Tau)	0.256*	-0.132*	-0.284*	-0.26*	0.24*	-0.282*	-0.235*
PET SUVR (FDG)	-0.37*	0.267*	0.399*	0.352*	-0.33*	0.319*	0.315*
Left_Hippocampus	-0.331*	0.272*	0.343*	0.187*	-0.416*	0.374*	0.237*

The ρ is the spearman correlation coefficients. Asterisk (*) next to r value indicates statistical significance ($p < 0.05$). NA, not applicable; CSF, cerebro-Spinal Fluid; Aβ, amyloid beta; p-Tau, phosphorylated tau 181; T-Tau, total tau; NfL, neurofilament light; PET, positron emission tomography; SUVR, standardized uptake value ratio; FDG, fluorodeoxyglucose; ADAS, Alzheimer's disease assessment scale-cognitive subscale; MMSE, mini-mental state examination; CDRSB, clinical dementia rating sum of boxes; ADNI-MEM, composite memory score; ADNI-EF, composite executive functioning score. ADAS-11 change, the average every-year decline of ADAS-11 points in 4 years; ADNI-MEM change, the average every-year decline of ADNI-MEM points in 4 years; ADNI-EF change, the average every-year decline of ADNI-EF points in 4 years.

Supplementary Table 12: Correlation of ATN biomarkers with memory tests among MCI & AD

ATN biomarkers (ρ)	ADAS-11	MMSE	ADNI-MEM	ADNI-EF	ADAS-11 change	ADNI-MEM change	ADNI-EF change
**CSF ATN biomarkers**							
CSF_A$\beta_{42/40}$	-0.305*	0.291*	0.368*	0.224*	-0.351*	0.35*	0.193*
CSF p-tau	0.34*	-0.312*	-0.39*	-0.29*	0.412*	-0.391*	-0.244*
CSF T-tau	0.326*	-0.303*	-0.368*	-0.282*	0.403*	-0.381*	-0.245*
CSF_NFL	0.271*	-0.21*	-0.301*	-0.299*	0.207*	-0.17*	-0.179*
**Plasma ATN biomarkers**							
Plasma_A$\beta_{42/40}$	-0.036	-0.033	0.047	0.082	0.013	0.033	0.052
Plasma p-tau	0.319*	-0.247*	-0.397*	-0.27*	0.305*	-0.322*	-0.241*
Plasma T-tau	0.196*	-0.131*	-0.191*	-0.178*	0.203*	-0.161*	-0.12*
Plasma_NFL	0.326*	-0.297*	-0.351*	-0.332*	0.273*	-0.24*	-0.213*
**Neuroimaging ATN biomarkers**							
PET SUVR(Aβ)	0.381*	-0.352*	-0.428*	-0.305*	0.405*	-0.427*	-0.303*
PET SUVR (Tau)	0.456*	-0.389*	-0.517*	-0.425*	0.498*	-0.5*	-0.374*
PET SUVR (FDG)	-0.555*	0.496*	0.578*	0.53*	-0.548*	0.498*	0.486*
Left_Hippocampus	-0.361*	0.301*	0.372*	0.14*	-0.396*	0.379*	0.158*

The ρ is the spearman correlation coefficients. Asterisk (*) next to r value indicates statistical significance ($p < 0.05$). NA, not applicable; CSF, cerebro-Spinal Fluid; Aβ, amyloid beta; p-Tau, phosphorylated tau 181; T-Tau, total tau; NfL, neurofilament light; PET, positron emission tomography; SUVR, standardized uptake value ratio; FDG, fluorodeoxyglucose; ADAS, Alzheimer's disease assessment scale-cognitive subscale; MMSE, mini-mental state examination; CDRSB, clinical dementia rating sum of boxes; ADNI-MEM, composite memory score; ADNI-EF, composite executive functioning score. ADAS-11 change, the average every-year decline of ADAS-11 points in 4 years; ADNI-MEM change, the average every-year decline of ADNI-MEM points in 4 years; ADNI-EF change, the average every-year decline of ADNI-EF points in 4 years.

Supple Table 13: Influence of of ATN biomarkers on cognitive performance and decline by ATN status for 4-year follow-up

ATN Group	ADAS-11			ADNI-MEM			ADNI-EF		
	Num	Baseline	Slope	Num	Baseline	Slope	Num	Baseline	Slope
**CSF**									
A-	394	6.00 [4.00;8.33]	0.04 [-0.29;0.62]	394	0.77±0.7	-0.02±0.11	394	0.88±0.89	-0.02±0.09
A+	264*	10.0 [6.67;14.0]	1.11 [0.09;2.47]	264*	0.16±0.78	-0.12±0.14	264*	0.25±1.06	-0.1±0.12
T-	401	6.33 [4.00;9.00]	0.07 [-0.31;0.62]	401	0.76±0.71	-0.02±0.11	401	0.86±0.89	-0.03±0.09
T+	256*	9.00 [6.00;14.3]	1.07 [0.07;2.44]	256*	0.15±0.78	-0.12±0.13	256*	0.26±1.06	-0.09±0.11
N-	214	8.67 [5.67;13.3]	0.58 [-0.05;2.19]	214	0.26±0.83	-0.09±0.12	214	0.16±0.91	-0.09±0.12
N+	185*	12.7 [9.67;17.7]	2.10 [0.53;3.61]	185*	-0.32±0.76	-0.17±0.12	185*	-0.43±0.99	-0.15±0.12
**Plasma**									
A-	218	10.7 [6.00;14.3]	0.90 [-0.10;2.96]	218	0.19±0.84	-0.1±0.14	218	0.13±1.03	-0.11±0.14
A+	514	10.7 [6.75;15.2]	1.13 [0.08;2.86]	514	0.02±0.89	-0.13±0.13	514	-0.09±0.99	-0.11±0.13
T-	397	6.67 [4.00;10.0]	-0.03 [-0.37;0.54]	397	0.77±0.76	-0.02±0.10	397	0.65±0.91	-0.02±0.10
T+	791*	9.00 [6.00;13.7]	0.56 [-0.12;2.25]	791*	0.26±0.87	-0.1±0.13	791*	0.21±1.00	-0.08±0.12
N-	662	7.00 [5.00;10.3]	0.14 [-0.32;0.95]	662	0.6±0.83	-0.05±0.12	662	0.51±0.96	-0.04±0.11
N+	917*	11.0 [7.00;16.7]	1.20 [0.10;3.12]	917*	-0.01±0.9	-0.13±0.13	917*	-0.09±1.05	-0.11±0.12
**Neuroimaging**									
A-	163	6.67 [4.00;9.00]	0.10 [-0.27;0.55]	163	0.71±0.78	-0.02±0.09	163	0.55±0.9	-0.03±0.09
A+	960*	8.00 [5.00;13.0]	0.46 [-0.17;1.79]	960*	0.37±0.91	-0.08±0.13	960*	0.34±1.06	-0.07±0.11
T-	358	5.67 [3.67;8.00]	0.07 [-0.33;0.62]	358	0.84±0.67	0.00±0.10	358	0.97±0.9	-0.03±0.07
T+	180*	10.7 [6.67;16.3]	1.16 [0.19;2.71]	180*	0.05±0.85	-0.13±0.12	180*	0.08±1.08	-0.09±0.08
N-	942	7.00 [4.33;9.67]	0.03 [-0.33;0.65]	942	0.6±0.73	-0.04±0.11	942	0.57±0.87	-0.04±0.10
N+	567*	11.3 [7.33;16.7]	1.49 [0.26;3.18]	567*	-0.34±0.8	-0.18±0.12	567*	-0.45±1.02	-0.15±0.11

Participants can be divided into A+ and A-, T+ and T-, N+ and N- with related biomarkers in CSF, Plasma and Neuroimaging higher or lower than the threshold value;

Baseline cognitive scores and cognitive annual changes were used to examine the influence of higher/lower ATN biomarkers on cognitive performances and decline over time, adjusted for age, gender, years of education; Results are mean ± SE or median [25th percent, 75th percent] according to normal distribution or not. *$p < 0.05$ compared to the negative status in slope. Num, number of participants; CSF, cerebro-Spinal Fluid; ADAS, Alzheimer's disease assessment scale-cognitive subscale; ADNI-MEM, composite memory score; ADNI-EF, composite executive functioning score.
